# Two Decades of Research on Marine-Derived *Alternaria*: Structural Diversity, Biomedical Potential, and Applications

**DOI:** 10.3390/md23110431

**Published:** 2025-11-07

**Authors:** Diaa T. A. Youssef, Areej S. Alqarni, Lamiaa A. Shaala, Alaa A. Bagalagel, Sana A. Fadil, Abdelsattar M. Omar, Mostafa E. Rateb

**Affiliations:** 1 Department of Natural Products, Faculty of Pharmacy, King Abdulaziz University, Jeddah 21589, Saudi Arabia; alqarniareej@gmail.com (A.S.A.); safadil@kau.edu.sa (S.A.F.); 2 King Fahd Medical Research Center, King Abdulaziz University, Jeddah 21589, Saudi Arabia; asmansour@kau.edu.sa; 3 Suez Canal University Hospitals, Suez Canal University, Ismailia 41522, Egypt; lamiaa.elnady@med.suez.edu.eg; 4 Department of Pharmacy Practice, Faculty of Pharmacy, King Abdulaziz University, Jeddah 21589, Saudi Arabia; abagalagel@kau.edu.sa; 5 Department of Pharmaceutical Chemistry, Faculty of Pharmacy, King Abdulaziz University, Jeddah 21589, Saudi Arabia; 6 School of Computing, Engineering and Physical Sciences, University of the West of Scotland, Paisley PA1 2BE, UK

**Keywords:** marine-derived *Alternaria*, bioactive compounds, cytotoxic, antimicrobial, anti-inflammatory, antidiabetic, phytotoxicity, antioxidant, antiparasitic

## Abstract

Marine-derived species of the genus *Alternaria* are widely distributed across diverse aquatic habitats, functioning as pathogens, endophytes, and saprophytes. These fungi are notable for their ability to produce structurally diverse secondary metabolites with potent bioactivities. Between 2003 and 2023, a total of 67 marine-derived *Alternaria* species were reported and investigated, collectively yielding 319 compounds. Most of these fungal isolates were from Chinese marine territories (53 species; ~79%), followed by isolates from Korea, Japan, India, Egypt, Saudi Arabia, and oceanic regions such as the Atlantic and Pacific. The fungal isolates were mainly obtained from marine plants (26 isolates) and marine animals (23 isolates), with additional sources including sediments (13) and seawater (3). Among the metabolites investigated in different screens, approximately 56% demonstrated measurable bioactivities, with anti-inflammatory (51 active compounds), antimicrobial (41 compounds), cytotoxic (39 compounds), and phytotoxic (52 compounds) activities being the most frequently reported. Additionally, compounds with antiparasitic, antidiabetic and antioxidant effects are reported. The chemical diversity of *Alernaria*-derived compounds spans multiple structural groups, including nitrogenous compounds, steroids, terpenoids, pyranones, quinones, and phenolics. Notably, compounds such as alternariol, alternariol monomethyl ether, and alternariol-9-methyl ether exhibit broad pharmacological potential, including antibacterial, antifungal, antiviral, immunomodulatory, and anticancer effects. Several metabolites also modulate cytokine production (e.g., IL-10, TNF-α), underscoring their relevance as immunomodulatory agents. Taken together, marine-derived *Alternaria* compounds represent a prolific and underexplored source of structurally and biologically diverse secondary metabolites with potential applications in drug discovery, agriculture, and biotechnology. This review provides an updated and comprehensive overview of the chemical and biological diversity of *Alternaria* metabolites reported over the past two decades, emphasizing their biomedical relevance and potential to inspire further research into their ecological functions, biosynthetic mechanisms, and industrial applications.

## 1. Introduction

The world’s oceans, covering more than 70% of the Earth’s surface, harbor an immense diversity of life, ranging from microscopic plankton to complex marine organisms. Among these, marine microbes, including fungi, play essential ecological roles and serve as prolific sources of bioactive secondary metabolites. Occupying diverse niches such as mangroves, sediments, sponges, algae, and corals, marine fungi often engage in symbiotic or competitive interactions, leading to the production of unique metabolites with significant biological activities. These distinctive compounds make marine fungi valuable resources for natural product discovery.

Marine fungi contribute substantially to marine ecosystems by cycling nutrients and providing energy to other organisms. Their secondary metabolites encompass a wide array of chemical classes, including polyketides, terpenoids, and alkaloids [1,2]. The genus *Alternaria* is particularly widespread, inhabiting multiple terrestrial and marine environments. Numerous secondary metabolites have been isolated from *Alternaria* species, exhibiting diverse biological activities such as antifungal, antibacterial, antiviral, and anticancer effects. Many of these compounds also display antioxidant and immunomodulatory properties [3]. Environmental factors, including temperature and light, can influence the biosynthesis of these metabolites, suggesting their potential use as biomarkers for monitoring marine environmental conditions.

The chemical diversity of *Alternaria* secondary metabolites is closely linked to the producing species. Some species generate cyclic polyketides, whereas others produce linear terpenoids, reflecting structural and functional variability [4]. This structural diversity underlies a broad spectrum of biological activities, making these compounds attractive candidates for drug discovery. Beyond pharmaceuticals, they hold promise for applications in food, cosmetics, agriculture, and environmental monitoring, such as natural insecticides, antifungal agents, or bioindicators. Further investigation into their structures and bioactivities is expected to reveal additional applications for these intriguing marine-derived compounds.

*Alternaria* species are ubiquitous in the environment, with spores commonly present in soil and air worldwide [5]. In addition to their ecological presence, exposure to *Alternaria* spores can trigger allergic reactions and asthma in sensitive individuals. Members of this genus are also important sources of phytotoxins, which can be harmful to their host organisms but exhibit diverse bioactivities [6]. While numerous reports document marine-derived *Alternaria* species, comprehensive, up-to-date information on their secondary metabolites and associated biological activities remains limited. Accordingly, this study provides an extensive overview of 319 secondary metabolites produced by 67 marine-derived *Alternaria* species studied between 2003 and 2023, with their biological activities serving as the basis for classification.

To record the data for this review, we performed a systematic literature search covering the period of 2003–2023. We queried databases including Web of Science, SciFinder, Scopus, and Google Scholar using the keywords “Alternaria”, “Marine Alternaria”, and “Marine-derived Metabolite”. A total of 208 hits, with some duplications, were found. Only manuscripts with reports about the isolation of new/novel compounds are considered in this review. Synthetic, biosynthetic, screening, and ecological studies are acknowledged where relevant but not explored in depth within this review. Also, duplicate reports and purely terrestrial studies were excluded. In total, 67 marine-derived *Alternaria* species and 319 compounds met our criteria. We note that our dataset may have a bias toward regions and sources that have been heavily studied (e.g., Chinese marine habitats), and some compounds (especially from less-accessible literature) could have been missed despite our efforts. We have addressed potential selection biases by cross-checking multiple sources and reviewing prior comprehensive articles on *Alternaria* metabolites to ensure coverage of known compounds. All structural representations assume the accuracy of assignments as reported in the original publications (we highlight where stereochemistry was later corrected or uncertain). Bioactivities were cataloged as “reported active” if the original study found a measurable activity (e.g., an IC_50_ or clear inhibition) in any assay; we did not impose a uniform potency cut-off, so “active” simply reflects a positive result in at least one biological test as per the source literature.

## 2. Reported Secondary Metabolites with Biological Properties

### 2.1. Compounds with Cytotoxic Activity (Table 1)

**Figure 1:**
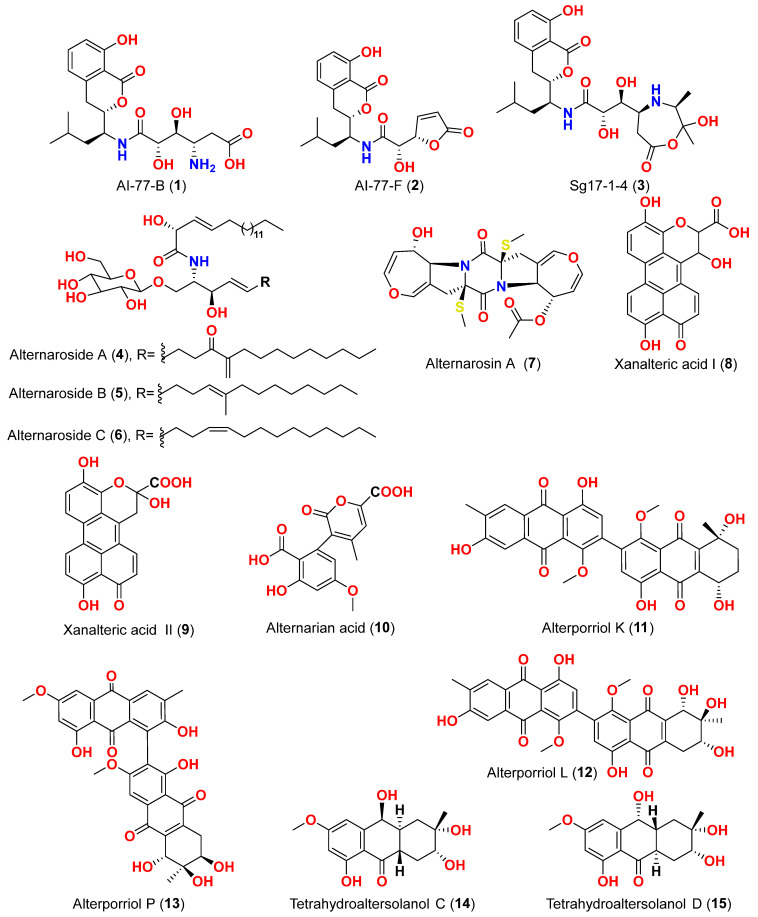
Structures of compounds **1**–**15**.

**Figure 2:**
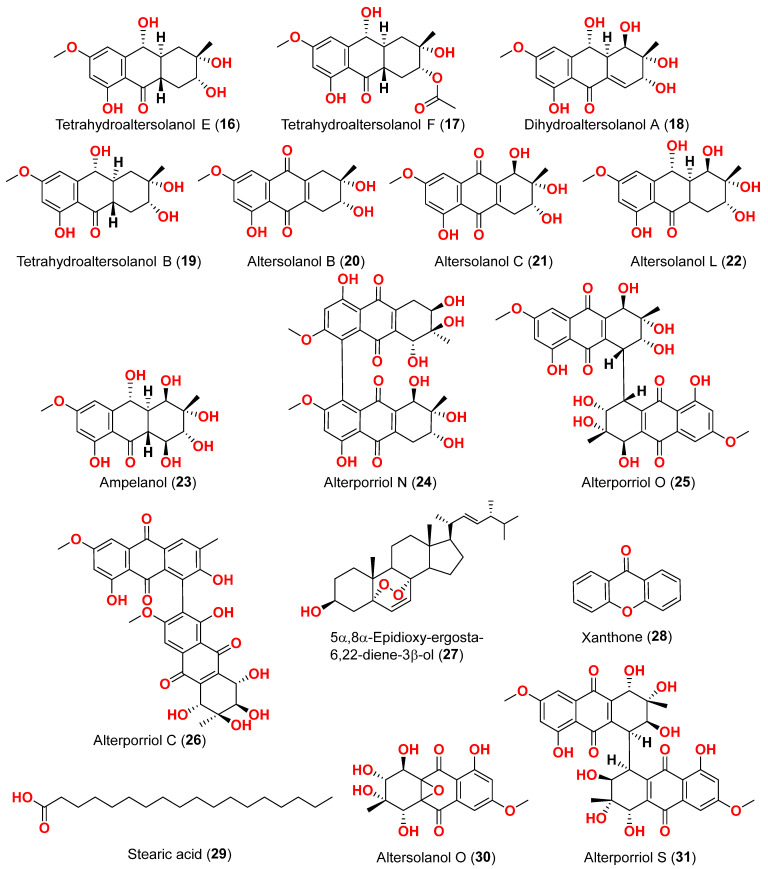
Structures of compounds **16**–**31**.

**Figure 3:**
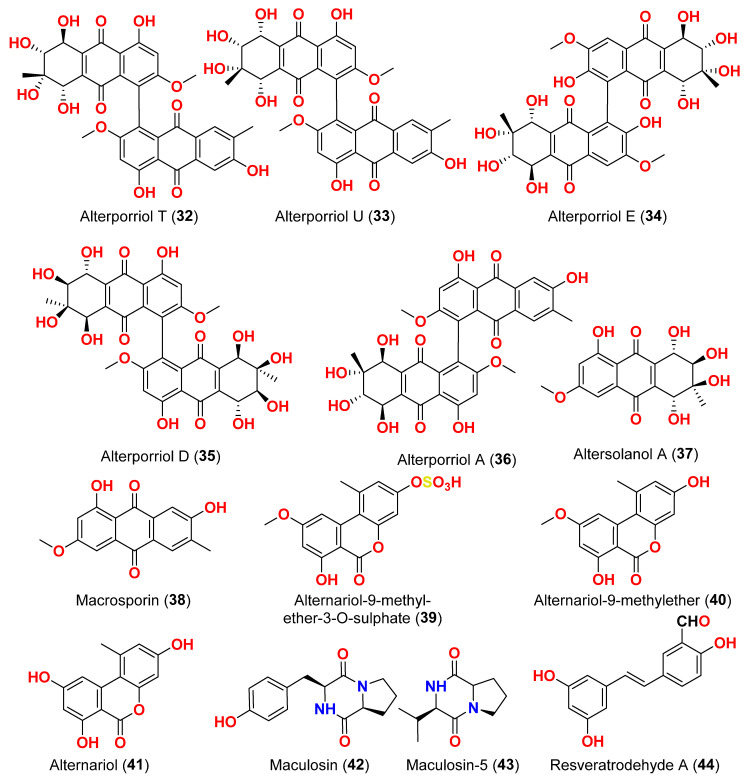
Structures of compounds **32**–**44**.

**Figure 4:**
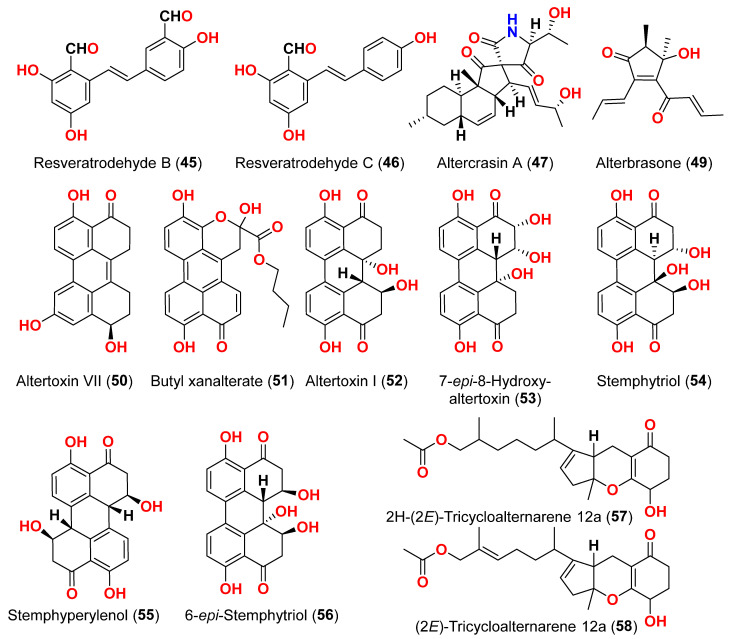
Structures of compounds **45**–**47**, **49**–**58**.

**Figure 5:**
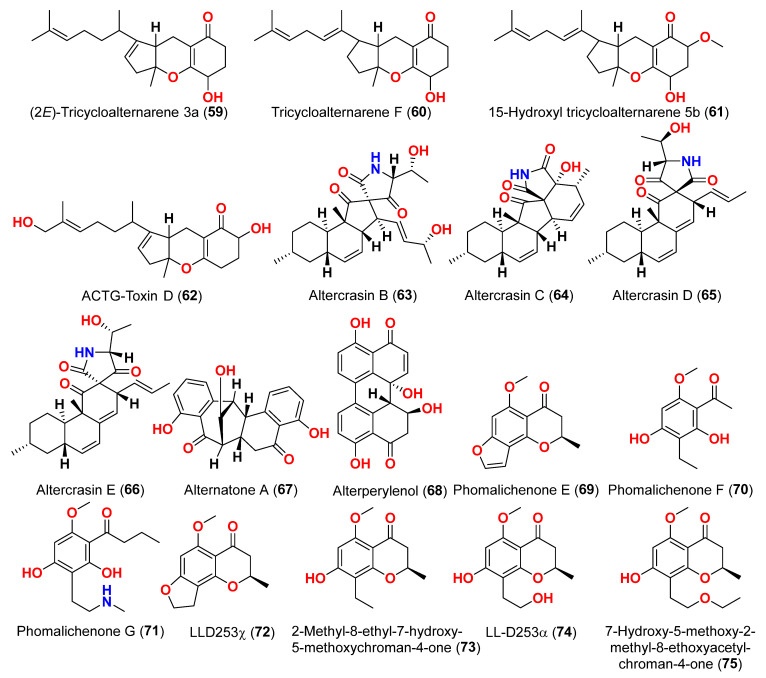
Structures of compounds **59**–**75**.

**Figure 6:**
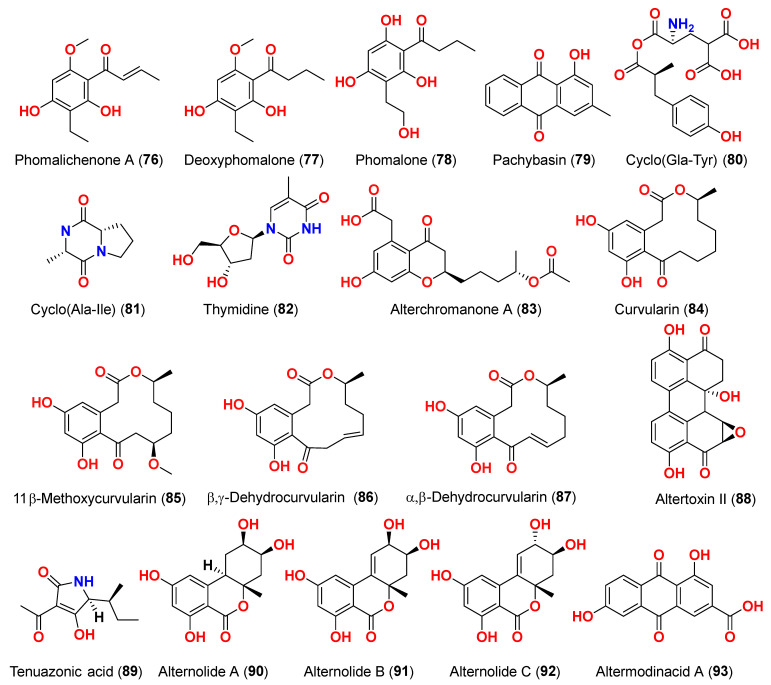
Structures of compounds **76**–**93**.

Different natural compounds reported from marine-derived *Alternaria* species have exhibited varying degrees of cytotoxic activity. In total, 93 compounds (**1**–**93**) (Figure 1, Figure 2, Figure 3, Figure 4, Figure 5 and Figure 6) have been identified and evaluated for their cytotoxic or growth-inhibitory effects against various cancer cell lines using diverse screening platforms, with or without reference control drugs [7,8,9,10,11,12,13,14,15,16,17,18,19,20,21,22,23,24,25,26,27,28,29,30,31]. Two isocoumarins, AI-77-B (**1**) and AI-77-F (**2**), with isocoumarin Sg17-1-4 (**3**), were yielded from the fungus *Alternaria tenuis* Sg17-1-4 obtained from a marine alga collected on Zhoushan Island, China [7,8]. In the MTT assay, cytotoxicity against human malignant A375-S2 and human cervical cancer Hela cells was assessed. AI-77-B (**1**) showed IC_50_ values of 100 and 20 µM against these cells, demonstrating potent activity. The IC_50_ values for Sg17-1-4 (**3**) were 300 and 50 µM, while AI-77-F (**2**) showed weak activity on Hela cells, with an IC_50_ value of 400 µM [7,8]. The marine-derived fungus *Alternaria raphani* (THW-18) was isolated from sediment collected in the Qingdao Sea salt field, Qingdao, China [9]. The cerebrosides alternarosides A–C (**4**–**6**) and the diketopiperazine alkaloid alternarosin A (**7**) were isolated from this fungus. The SRB method was used to measure cytotoxicity against cancer cells P388 and HL-60, while the MTT method was used to measure cytotoxicity against a cancer cell A549 and a normal cell BEL-7402. No cytotoxic effect was observed in the four cancer cell lines (IC_50_ > 100 μM) [9]. Extracts of the fungus *Alternaria* sp. JCM9.2, isolated from the mangrove *Sonneratia alba* collected in the Dong Zhai Gang Mangrove Garden on the Chinese island of Hainan, generated three carboxylic acids: xanalteric acids I (**8**) and II (**9**) and alternarian acid (**10**). Using the MTT test, the cytotoxicity of these compounds against L5178Y cells was determined. However, none of the compounds displayed significant efficacy [10]. Alterporriols K and L (**11** and **12**) are dimeric bianthraquinone derivatives with C-2-C-2′ connections, obtained from extract fungus *Alternaria* sp. ZJ9-6B, obtained from mangroves of *Aegiceras corniculatum* collected in the South China Sea [11,12]. According to preliminary bioassays, compounds **11** and **12** have modest cytotoxic effects on human breast cancer cell lines. The IC_50_ values for compound **11** were 26.97 and 29.11 µM against MDA-MB-435 and MCF-7 cells, respectively. However, compound **12** reduced the development of MDA-MB-435 and MCF-7 with IC_50_ values of 13.11 and 20.04 µM, respectively. Furthermore, alterporriol L (**12**) showed significant inhibition of growth rate in both cell lines in a dose-dependent manner compared to control cells. More than 86% of cells were inhibited by compound **12** at a concentration of 50 µM [11,12]. Alterporriol P (**13**) is an anthraquinone dimer derivative, identified in China by culture of the endophytic fungus *Alternaria* sp. ZJ-2008003 from *Sarcophyton* sp. soft coral [13]. Alterporriol P (**13**) inhibited the proliferation of the human prostate cancer cell line PC-3 and the colon cancer cell line HCT-116 [13]. In contrast, the fungus *Alternaria* sp. ZJ-2008003, isolated from *Sarcophyton* sp., a soft coral in the South China Sea, afforded tetrahydroaltersolanols C-F (**14**–**17**), dihydroaltersolanol A (**18**), tetrahydroaltersolanol B (**19**), altersolanol B (**20**), altersolanol L (**22**), and ampelanol (**23**) with an oxidized C-10 and a reduced C-9 fragment, which were inert (IC_50_ > 100 μM) [14]. The anthraquinone derivative with a paraquinone group, altersolanol C (**21**), exhibited cytotoxicity against human colon carcinoma HCT-116, human breast cancer MCF-7/ADR, human prostatic cancer PC-3, and human hepatoma HepG2 and Hep3B cell lines with IC_50_ values ranging from 2.2 to 8.9 μM [14]. These results suggest that the presence of a paraquinone group is crucial for the observed cytotoxicity. Additionally, among the alterporriol-type dimers, alterporriols N, O, P (**24**, **25**, **13**), alterporriol P (**13**) showed cytotoxicity against PC-3 and HCT-116 with IC_50_ values of 6.4 and 8.6 μM, respectively. However, alterporriol C (**26**) was determined to be inactive (IC_50_ > 20 μM) [14].

The mycelium of the Chinese marine fungus *Alternaria* sp. MNP801 was extracted to produce three compounds including 5α,8α-epidioxy-ergosta-6,22-diene-3-ol (**27**), xanthone (**28**), and stearic acid (**29**). The IC_50_ values of compound **27** against H460, 3T3, PC12, and U937A tumor cells were 119.6, 96.2, 27.1, and 34.1 μM, respectively. 5α,8α-Epodioxyergosta-6,22-diene-3β-ol (**27**) was equally potent against H460 and 3T3 tumor cells and more effective than the VP16 in combating PC12 and U937A tumor cells [15]. The fungus *Alternaria* sp. XZSBG-1 was isolated from Salt Lake in Bange, Tibet, China. The anthraquinone derivatives, altersolanol C (**21**), alterporriol N (**24**), altersolanol O (**30**), alterporriol S (**31**), alterporriol T (**32**), alterporriol U (**33**), alterporriol E (**34**), alterporriol D (**35**), alterporriol A (**36**), altersolanol A (**37**), and macrosporin (**38**), were isolated and identified [16]. MTT assays were used to assess the cytotoxic activities of compounds **21**, **24,** and **30**–**38** against MCF-7/ADR, HeLa, and HCT-116 cell lines. Altersolanol C (**21**) exhibited potent inhibitory action against HCT-116 and HeLa cell lines, with IC_50_ values of 3.0 and 8.0 μM, respectively. The remaining compounds did not exhibit any significant inhibitory effect against the cancer cell lines examined [16]. A fraction of EtOAc extract recovered from the culture broth of the fungus *Alternaria alternata*, isolated from the Egyptian Red Sea soft coral *Litophyton arboretum*, afforded alternariol-9-methyl ether-3-*O*-sulphate (**39**), alternariol-9-methyl ether (**40**), alternariol (**41**), maculosin (**42**), and maculosin-5 (**43**) [17]. Using the disk diffusion test, all compounds were evaluated for anticancer activity against two leukemia cells (murine L1210 and human CCRFCEM), four solid cancers (murine colon 38, human colon HCT116, human lung H125, and human liver HEPG2), and a normal human cell (CFUGM). Bioassay revealed that compound **39** was marginally selective against solid tumor HEPG2 compared to normal human cells (CFUGM) when 3 μg/disk of alternariol-9-methyl ether-3-*O*-sulphate (**39**) was used. In contrast, the fungal extracts (30 μg/disk), alternariol-9-methyl ether (**40**) and alternariol (**41**) inhibited normal cell growth [17]. Three resveratrol derivatives, resveratrodehydes A–C (**44**–**46**), were obtained from the fungus *Alternaria* sp. R6, isolated from the mangrove *Myoporum bontioides* found in Guangdong Province, China [18]. These compounds exhibited inhibitory effects against breast MDA-MB-435, liver HepG2, and colon HCT-116 human cells, as determined by the MTT assay. The antitumor effects of the compounds exhibit superiority in vitro compared to the positive control, resveratrol at concentrations below 50 μM. Compounds **44** and **45** displayed significant cytotoxicity against both the MDA-MB-435 and HCT-116 cell lines, with IC_50_ values < 10 μM [18]. A decalin derivative, altercrasin A (**47**), with spiro skeletons isolated from a strain of *Alternaria* sp. OUPS-117D-1, was isolated from the sea urchin *Anthocidaris crassispina*, Japan [19]. Altercrasin A (**47**) inhibited human HL-60 leukemia and taurine L1210 leukemia cell lines with IC_50_ values of 21.5 and 22.1 µM, respectively [19]. Compound AS2-1 (**48**), a polysaccharide with a molecular weight of 27.4 kDa was isolated from the fungus *Alternaria* sp. SP-32, obtained from a sponge collected from the South China Sea [20]. AS2-1 (**48**) has a concentration-dependent cytotoxic effect on tested cell lines. The IC_50_ values of compound **48** in Hela, HL-60, and K562 cell lines using the MTT and SRB methods were 6.4, 5.2, and 16.7 μM, respectively [20]. Alterbrasone (**49**) was separated from the fungus *Alternaria brassicae* 93, isolated from crinoid *Comanthina schlegeli* collected from the South China Sea [21]. Cytotoxicity against two human cancer cell lines including human breast carcinoma cell line (MDA-MB-435) and human lung cancer cell line (A549) displayed no activity against these cells in the MTT assay [21].

Two derivatives of perylenequinone, altertoxin VII (**50**) and butyl xanalterate (**51**), as well as five compounds, altertoxin I (**52**), 7-*epi*-8-hydroxyaltertoxin (**53**), stemphytriol (**54**), stemphyperylenol (**55**), and 6-*epi*-stemphytriol (**56**), were isolated from the fungus *Alternaria* sp. SCSIO41014 derived from the sponge *Callyspongia* sp. collected from a coastal province in China [22]. Using the CCK-8 assay, the cytotoxic effects of these compounds against human erythroleukemia (K562), human gastric carcinoma (SGC-7901), and hepatocellular cancer cells (BEL-7402) were investigated. Paclitaxel as the positive control showed IC_50_ values of 0.21, 01.04, and 0.63 µM, respectively. Among the studied compounds, altertoxin VII (**50**) was cytotoxic to the K562, SGC-7901, and BEL-7402 cell lines with corresponding IC_50_ values of 82.6, 27.1, and 40.9 µM, respectively. Selective cytotoxic action against K562 with an IC_50_ of 53.2 µM was exhibited by 6-*epi*-stemphytriol (**56**) [22]. The fungus *Alternaria* sp. W-1 associated with the Chinese alga *Laminaria japonica* produced 2H-(2*E*)-tricycloalternarene 12a (**57**), as well as five analogs, (2*E*)-tricycloalternarene 12a (**58**), tricycloalternarene 3a (**59**), tricycloalternarene F (**60**), 15-hydroxyl tricycloalternarene 5b (**61**), and ACTG-Toxin D (**62**) [23]. The MTT assay evaluated cytotoxicity against the human hepatocellular carcinoma SMMC-7721 and the human gastric carcinoma SGC-7901 cell lines. Compounds 2H-(2*E*)-tricycloalternarene 12a (**57**), (2*E*)-tricycloalternarene 3a (**59**), and tricycloalternarene F (**60**) decreased SMMC-7721 cell growth with corresponding IC_50_ values of 127.4, 138.7, and 243.3 µM, while cisplatin had an IC_50_ value of 21.5 µM. (2*E*)-Tricycloalternarene 3a (**59**) and ACTG-Toxin D (**62**) exhibited a moderate antiproliferation action against SGC-7901 cells, with IC_50_ values of 15.7 and 101.4 µM, respectively, compared to the IC_50_ value of cisplatin of 14.9 µM. Further analysis revealed that the anticancer action of (2*E*)-tricycloalternarene 3a (**59**) against SMMC-7721 cells was related to G1 phase inhibition and cell apoptosis, using both the mitochondrial and death receptor pathways [23]. The fungus *Alternaria* sp. OUPS-117D-1, which was isolated from the sea urchin *Anthocidaris crassispina* from Japan, developed four decalin derivatives, classified as altercrasins B–E (**63**–**66**) [24]. The chemical pairings altercrasin B/altercrasin C (**63**/**64**) and altercrasin D/altercrasin E (**65**/**66**) were determined to be respective stereoisomers. The cytotoxic actions of altercrasins B-E (**63**–**66**) and 5-fluorouracil were investigated. Consequently, their cytotoxicity against murine L1210 leukemia, murine P388 leukemia, and human HL-60 leukemia cell lines revealed that compounds **65** and **66,** containing a diene moiety (C-6 to C-8), exhibited strong cytotoxic activity against these cancer cells, especially the HL-60 cell line. In particular, the activity of compound **65** was comparable to that of 5-fluorouracil [24]. Alternatone A (**67**), having an unusual tricyclo[6.3.1.02,7]dodecane structure, was isolated from a soft coral-derived fungus *Alternaria alternata* L3111′, along with three known perylenequinones, altertoxin I (**52**), stemphyperylenol (**55**), and alterperylenol (**68**) [25]. All compounds were exposed to a cytotoxic activity evaluation against human lung carcinoma (A-549), human colon cancer (HCT-116), and human cervical carcinoma (HeLa) cell lines. Alterperylenol (**68**) exhibited cytotoxicity against A-549, HCT-116, and HeLa cell lines with corresponding IC_50_ values of 2.6, 2.4, and 3.1 μM, respectively. However, the remaining compounds did not display cytotoxic actions. This demonstrates how double bonds in perylenequinones are crucial for their cytotoxicity [25].

Three phomalichenones E-G (**69**–**71**) and seven analogs, including LL-D253γ (**72**), 2-methyl-8-ethyl-7-hydroxy-5-methoxychroman-4-one (**73**), LL-D253α (**74**), 7-hydroxy-5-methoxy-2-methyl-8-ethoxyacetylchroman-4-one (**75**), phomalichenone A (**76**), deoxyphomalone (**77**), and phomalone (**78**) originated from an *Alternaria* sp. fungus MCCC 3A00467 isolated from deep sediment in the Pacific Ocean [26]. The MTT test was used to assess the cytotoxicity of compounds **69**–**78** against human myeloma cancer U266, human liver cancer (HepG2), and human lung cancer (A549) cells. Compounds **70**, **74**, and **76**–**78** inhibited the growth of U266 and HepG2 human cells, while phomalone (**78**) exhibited the highest cytotoxic action against three cancer cell lines, with IC_50_ values ranging from 55.0 to 60.8 µM. Based on IC_50_ values greater than 396.8–431.0 µM for compounds **69** and **72**–**75** against U266 and HepG2 cells, the number of hydroxyl groups can influence cytotoxicity [26]. Compounds **74** and **76**–**78** inhibited U266 and HepG2 cells with IC_50_ values between 55.0 and 256.7 µM, while phomalichenones E (**69**) and LL-D253γ (**72**) without hydroxyl groups are inactive (IC_50_ > 427.0–431.0 µM). LL-D253α (**74**) was more cytotoxic than compounds **73** and **75** against three cell lines, indicating that the hydroxyl group at C-2′ may play an essential role in antitumor action. Comparing the IC_50_ values of compounds **74** and **78** revealed that the open pyrone ring or the presence of phenolic hydroxyl group at position C-2 had no impact on the activity against U266 and HepG2 cells but had a substantial effect on A549 cells. Compounds **70** and **78** demonstrated that the methylamino group at C-2′ decreased the inhibitory effect [26]. Through investigation of the fungal extract of *Alternaria* sp. 114-1G, compounds pachybasin (**79**), cyclo(Gla-Tyr) (**80**), cyclo(Ala-Ile) (**81**), and thymidine (**82**) were purified and identified [27]. The most efficient inhibitory impact on HeLa cells was observed by pachybasin (**79**) with a 57.8% inhibition rate at 420.1 µM [27]. The fungus *Alternaria longipes*, isolated from the mangrove of *Kandelia candel* in Guangxi, China, afforded one chromanone derivative, alterchromone A (**83**), and four curvularin-type macrolides curvularin (**84**), 11-β-methoxycurvularin (**85**), β,γ-dehydrocurvularin (**86**), and α,β-dehydrocurvularin (**87**) [28]. Compounds **83**–**87** were evaluated for their cytotoxicity against four human tumor cell lines, including HeLa (human cervical carcinoma cell line), HepG2 (human hepatocellular carcinoma cell line), MCF-7 (human breast cancer cell line), and ACHN (human renal carcinoma cell line). Interestingly, the 100 μM concentration of these chemicals had no detectable inhibitory impact on the tested cell lines [28].

Isolation of five polyketides, alternariol (**41**), alternariol-9-methyl ether (**40**), altertoxin I (**52**), altertoxin II (**88**), and tenuazonic acid (**89**), from the marine endophytic fungus *Alternaria* sp. LV52 isolated from *Cystoseira tamariscifolia* collected from the Red Sea, Egypt, was reported [29]. However, all compounds exhibited cytotoxicity against HepG2 with corresponding EC_50_ values ranging from 27.8 to 172.0 μM. The cytotoxicity of alternariol-9-methyl ether (**40**), altertoxin II (**88**), and tenuazonic acid (**89**) was evaluated against A549 and PC3 cells [29]. Thus, the EC_50_ of alternariol-9-methyl ether (**40**) and altertoxin II (**88**) were 1.43 and 1.14 μM against A549 and 0.65 and 0.34 μM against PC3, respectively. Tenuazonic acid (**89**) showed moderate activity against A549 and PC3 cells. Moreover, compound **89** was the only chemical identified as cytotoxic for the HeLa cell line with an EC_50_ value of 109.1 μM [29]. The fungus *Alternaria alternata* LW37, a marine-derived fungus obtained from deep-sea sediment on the southwest Indian Ridge, produced three dibenzo-α-pyrone compounds, alternolides A–C (**90**–**92**). The cytotoxicity of compounds **90**–**92** was assessed against MCF-7 (human breast cancer cells), B16 (mouse melanoma cells), and HepG2 (human hepatocellular carcinoma cells). The compounds exhibited no detectable inhibitory effect on the investigated cell lines at 50 µM [30]. The cytotoxic activity of anthraquinone, altermodinacid A (**93**), was assessed following its discovery in the fungus *Alternaria* sp. X112, which was isolated from a marine fish *Gadus macrocephalus* from Yangma Island, China. No cytotoxic effects (IC_50_ > 40 µM) were observed against MCF-7, MKN-45, TE-1, and HCT116 cells [31].

In conclusion, from the above discussion and from the data presented in Table 1, perylenequinones, altertoxins, and altersolanols represent the most cytotoxic agents among the evaluated compounds in this section.

Among the 93 evaluated compounds, findings were as follows: Perylenequinones and altertoxins: Altertoxins I (**52**) and II (**88**) exhibited strong cytotoxicity against A549 and PC3 cells, with EC_50_ values as low as 0.34–1.14 μM, while altertoxins also showed activity against HepG2 cells at slightly higher EC_50_ values (42.8–131.7 μM) [29]. Alternariol derivatives: Alternariol (**41**) and alternariol-9-methyl ether (**40**) were cytotoxic against HepG2, HeLa, A549, and PC3 cells, with EC_50_ values as low as 0.65 μM (PC3) and 1.43 μM (A549) [29]. Altersolanols: Altersolanol C (**21**) showed broad cytotoxicity across HCT-116, MCF-7/AD, PC-3, HepG2, and Hep3B cells, with IC_50_ values of 2.2–8.9 μM. Additional assays with *A.* sp. XZSBG-1 confirmed strong cytotoxicity against MCF-7/ADR, HeLa, and HCT-116 cells (IC_50_ = 3.0–8.0 μM) [14,16]. Resveratrodehydes: Resveratrodehydes A–C (**44**–**46**) exhibited potent cytotoxicity against MDA-MB-435, HepG2, and HCT-116 cells, with IC_50_ values ranging from 6.9 to 18.6 μM [18]. Alterporriols: Alterporriol P (**13**) showed strong inhibitory effects against PC-3 and HCT-116 cells (IC_50_ = 6.4 and 8.6 μM) [13]. Alterporriols K (**11**) and L (**12**) demonstrated notable cytotoxicity against MDA-MB-435 and MCF-7 cells, with IC_50_ values of 26.9–29.1 μM and 13.1–20.0 μM, respectively; alterporriol L killed 86% of cells at 50 μM [11,12].

Among the moderately active compounds are compound AI-77-B (**1**), which displayed IC_50_ values of 20 and 100 μM (Hela and A375-S2), while AI-77-F (**3**) was less active (IC_50_ = 50 and 300 μM) [7,8]. Xanalteric acids I (**8**) and II (**9**) exhibited IC_50_ values of 45.0 and 87.5 μM against murine L5178Y cells [10]. Altercrasin A (**47**) and polysaccharide AS2-1 (**48**) showed IC_50_ values of 21.5–22.1 μM and 5.2–16.7 μM, respectively, against leukemia and HeLa cells [19,20]. Altertoxin VII (**50**) exhibited IC_50_ values of 27.1–82.8 μM against K562, SGC-7901, and BEL-7402 cells [22].

Weakly active compounds included 6-epi-stemphytriol (**56**) with an IC_50_ of 53.2 μM (K562), while tricycloalternarenes **57**–**60** and ACTG-toxin D (**62**) displayed IC_50_ values >100 μM [23]. Altercrasins B–E (**63**–**66**) exhibited IC_50_ values ranging from 15.3 to 165.8 μM, depending on the cell line [24]. Phomalichenones (**70**, **76**), deoxyphomalone (**77**), and phomalone (**78**) showed IC_50_ values between 55.0 and 381.9 μM [26]. Pachybasin (**79**) inhibited 57.8% of HeLa cells at 420.1 μM [27]. Tenuazonic acid (**89**) exhibited weaker activity, with EC_50_ values above 100 μM in HepG2 and HeLa cells.

Overall, compounds based on perylenequinone, altertoxin, and altersolanol scaffolds represent the most potent cytotoxins, frequently exhibiting IC_50_/EC_50_ values below 10 μM. Other structural classes, including tetramic acid derivatives, diphenyl ethers, and ergosterol-type compounds, show moderate to weak cytotoxic effects. Most reported metabolites fall within the moderate range (50–100 μM), suggesting opportunities for structure optimization to improve potency and selectivity.

Cytotoxicity Mechanistic Insight. Many cytotoxic *Alternaria* metabolites, especially perylenequinones (e.g., altertoxins, stemphyperylenol, alterperylenol), are known to act as photosensitizers that generate reactive oxygen species (ROS) under light, contributing to cell damage [32]. The production of ROS leading to oxidative stress is believed to play a role in their anticancer effects, as demonstrated by altertoxins encouraging lipid peroxidation in cell membranes [15]. Other compounds, like alternariol (**41**) and alternariol monomethyl ether (**138**), have been shown to intercalate DNA and inhibit eukaryotic topoisomerase enzymes, leading to DNA strand breaks in cancer cells [16]. The relatively planar structures of these anthraquinones facilitate such interactions. It is also notable that slight structural modifications can alter potency: e.g., alternariol and its methyl ether differ in activity, suggesting that the hydroxylation pattern on the aromatic rings influences their ability to bind DNA or other targets. However, beyond a few cases studied, detailed pharmacological target data are lacking for most *Alternaria* cytotoxins, representing an area for future research (See Conclusions and Future Trends). Table 1 shows only the compounds with proven cytotoxic activity.

**Table 1:** Reported compounds with proven cytotoxic activities.

Compound	Cell Line Used	Biological Activity	Fungus Name	Host Organism	Reference
AI-77-B (**1**)	A375-S2, HeLa	IC_50_ = 100, 20 μM	*Alternaria tenuis*, Sg17-1	Unspecified alga	[7,8]
AI-77-F (**2**)		IC_50_ = 400 μM (Hela)			
Sg17-1-4 (**3**)		IC_50_ = 300, 50 μM			
Xanalteric acid I (**8**)	L5178Y	IC_50_ = 45.0 μM	*Alternaria* sp. JCM9.2	Mangrove *Sonneratia alba*	[10]
Xanalteric acid II (**9**)		IC_50_ = 87.5 μM			
Alternarian acid (**10**)		IC_50_ = 99.2 μM			
Alterporriol K (**11**)	MDA-MB-435, MCF-7	IC_50_ = 26.9, 29.1 μM	*Alternaria* sp. ZJ9-6B	Mangrove *Aegiceras corniculatum*	[11,12]
Alterporriol L (**12**)		IC_50_ = 13.1, 20.0 μM 86% cells killed at 50 μM			
Alterporriol P (**13**)	PC-3, HCT-116	IC_50_ = 6.4, 8.6 μM (PC-3, HCT-116)	*Alternaria* sp. ZJ-2008003	*Sarcophyton* sp. soft coral	[13,14]
Altersolanol C (**21**)	HCT-116, MCF-7/AD, PC-3, HepG2, Hep3B	IC_50_ = 2.2–8.9 μM	*Alternaria* sp. ZJ-2008003	*Sarcophyton* sp. soft coral	[14]
	MCF-7/ADR, HeLa, HCT-116	IC_50_ = 3.0, 8.0 μM (HCT-116, HeLa)	*Alternaria* sp. XZSBG-1	Sediment	[16]
5α,8α-Epidioxy-ergosta-6,22-dien-3β-ol (**27**)	H460, 3T3, PC12, U937	IC_50_ = 119.6, 96.2, 20.3, 34.1 μM	*Alternaria* sp. MNP801		[15]
Alternariol-9-methyl ether-3-*O*-sulphate (**39**)	Two leukemias (L1210, CCRFCEM); four solid tumors (murine colon 38, HCT116, H125, HEPG2); one normal cell (CFU-GM)	400 zu against HEP-G2 compared to 100 zu against CFU-GM at 3 μg/disk	*Alternaria alternata*	Soft coral *Litophyton arboreum*	[17]
Alternariol-9-methyl ether (**40**)		Cytotoxic to CFU-GM			
	HepG2, Hela, A549, PC3	EC_50_ = 108.5 μM (HepG2) EC_50_ = 1.43 μM (A549) EC_50_ = 0.65 μM (PC3)	*Alternaria* sp. LV52	*Cystoseira tamariscifolia*	[29]
Alternariol (**41**)	CFU-GM	Cytotoxic to CFU-GM	*Alternaria alternata*	Soft coral *Litophyton arboreum*	[17]
	EC_50_ = 37.9 μM (HepG2)	EC_50_ = 37.9 μM (HepG2)	*Alternaria* sp. LV52	*Cystoseira tamariscifolia*	[29]
Resveratrodehyde A (**44**)	MDA-MB-435, HepG2, HCT-116	IC_50_ = 8.5, 7.8 μM (MDA-MB-435, HCT-116)	*Alternaria* sp. R6	Mangrove *Myoporum bontioides*	[18]
Resveratrodehyde B (**45**)		IC_50_ = 7.6, 6.9 μM (MDA-MB-435, HCT-116)			
Resveratrodehyde C (**46**)		IC_50_ = 16.4, 18.6 μM (MDA-MB-435, HCT-116)			
Altercrasin A (**47**)	Human HL-60 leukemia, taurine L1210 leukemia	IC_50_ = 21.5, 22.1 μM	*Alternaria* sp. OUPS-117D-1	Sea urchin *Anthocidariscrassispina*	[19]
Polysaccharide AS2-1 (**48**)	Hela, HL-60, K562	IC_50_ = 6.4, 5.2, 16.7 μM	*Alternaria* sp. SP-32	Unspecified sponge	[20]
Altertoxin VII (**50**)	K562, SGC-7901, BEL-7402	IC_50_ = 82.8, 27.1, 40.9 µM	*Alternaria* sp. SCSIO41014	*Callyspongia* sp. sponge	[22]
6-*epi*-Stemphytriol (**56**)		IC_50_ = 53.2 µM (K562)			
2H-(2*E*)-Tricycloalternarene 12a (**57**)	SMMC-7721, SGC-7901	IC_50_ = 127.4 µM (SMMC-7721)	*Alternaria* sp. W-1	Algae *Laminaria japonica*	[23]
(2*Z*)-Tricycloalternarene 3a (**59**)		IC_50_ = 138.7, 15.7 µM (SMMC-7721, SGC-7901)			
Tricycloalternarene F (**60**)		IC_50_ = 243.3 µM (SMMC-7721)			
ACTG-Toxin D (**62**)		IC_50_ = 101.4 µM (SGC-7901)			
Altercrasin B (**63**)	P388, HL-60, L1210	IC_50_ = 57.8, 29.1, 19.2 µM	*Alternaria* sp. OUPS-117D-1	*Urchin Anthocidaris crassispina*	[24]
Altercrasin C (**64**)		IC_50_ = 165.8, 112.7, 73.4 µM			
Altercrasin D (**65**)		IC_50_ = 24.4, 15.3, 21.1 µM			
Altercrasin E (**66**)		IC_50_ = 39.0, 15.6, 25.9 µM			
Phomalichenone F (**70**)	U266, HepG2, A549	IC_50_ = 118.5, 157.6, 381.9 µM	*Alternaria* sp. MCCC 3A00467	Deep ocean sediment	[26]
LL-D253α (**74**)		IC_50_ = 61.1, 132.9 µM (U266, HepG2)			
Phomalichenone A (**76**)		IC_50_ = 62.2, 66.5, 86.4 µM			
Deoxyphomalone (**77**)		IC_50_ = 98.3, 65.5, 256.7 µM			
Phomalone (**78**)		IC_50_ = 55.0, 60.8, 101.2 µM			
Pachybasin (**79**)	HeLa	57.8% inhibition rate at 420.1 µM	*Alternaria* sp. 114-1G	Ocean	[27]
Altertoxin I (**52**)	HepG2, Hela, A549, PC3	EC_50_ = 131.7 μM (HepG2)	*Alternaria* sp. LV52	*Cystoseira tamariscifolia*	[29]
Altertoxin II (**88**)		EC_50_ = 42.8 μM (HepG2) EC_50_ = 1.14 μM (A549) EC_50_ = 0.34 μM (PC3)			
Tenuazonic acid (**89**)		EC_50_ = 146.1 μM (HepG2) EC_50_ = 109.1 μM (HeLa)			

Notes: IC_50_ and EC_50_ values for all compounds have been converted to μM for consistency. Table 1 shows each compound only once; if a compound was reported in multiple studies, the most potent IC_50_ is listed, with additional data described in the text or footnotes.

### 2.2. Compounds with Antimicrobial Activity (Table 2)

**Figure 7:**
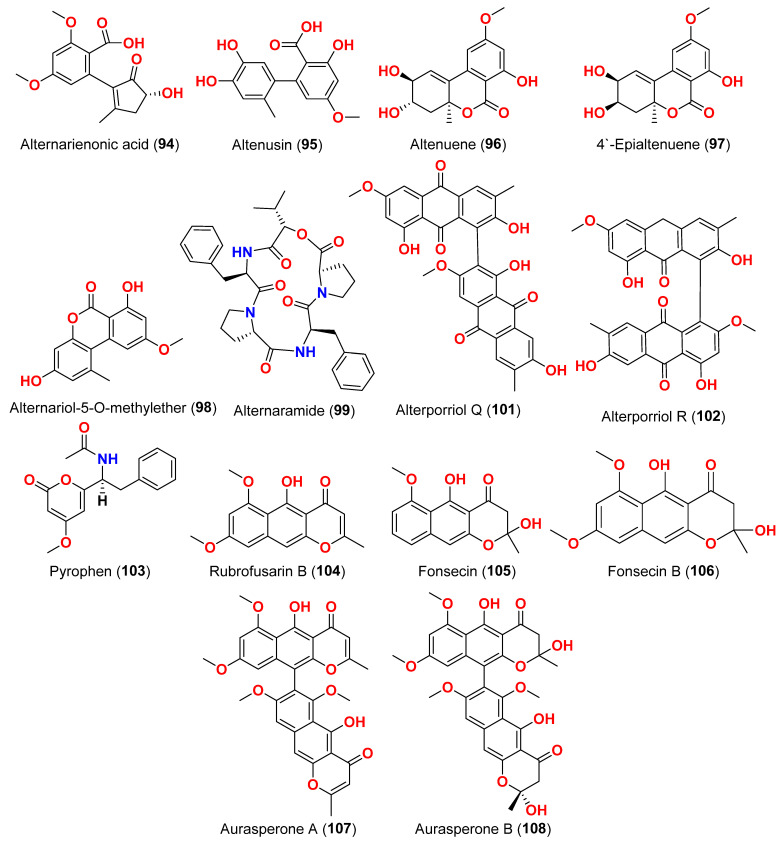
Structures of compounds **94**–**99**, **101**–**108**.

**Figure 8:**
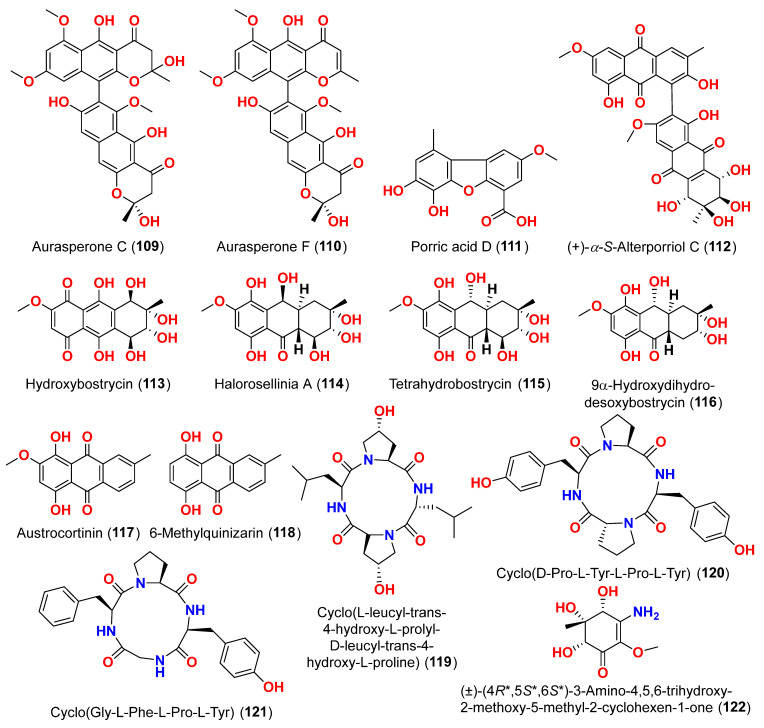
Structures of compounds **109**–**122**.

**Figure 9:**
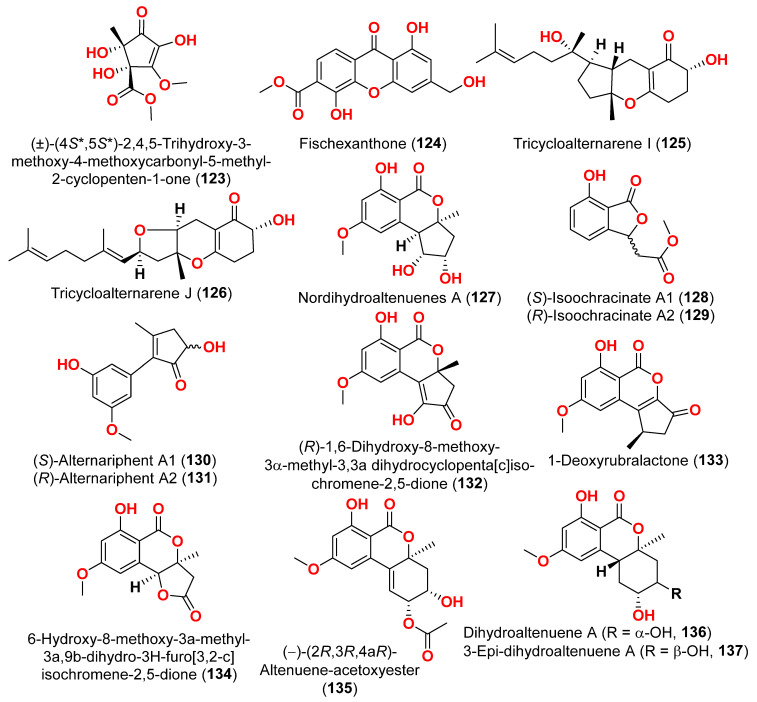
Structures of compounds **123**–**137**.

**Figure 10:**
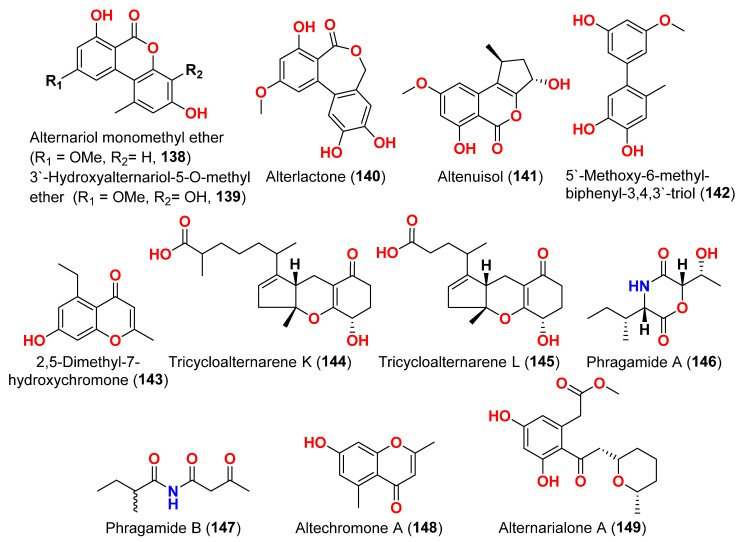
Structures of compounds **138**–**149**.

**Figure 11:**
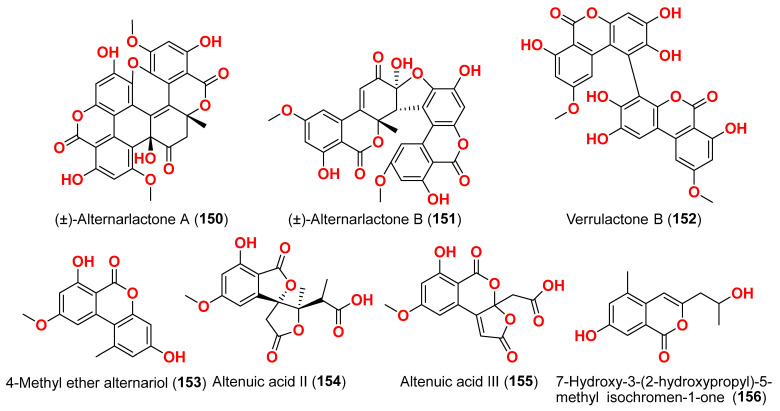
Structures of compounds **150**–**156**.

The secondary metabolites produced by members of the genus *Alternaria* are essential for survival in the marine environment. Consequently, they may have medicinal applications, including antibacterial, antifungal and antiviral activities. Compounds evaluated for their activity in this section are displayed in Figure 1, Figure 2, Figure 3, Figure 4, Figure 5, Figure 6, Figure 7, Figure 8, Figure 9, Figure 10 and Figure 11 and compounds with proven antimicrobial activity have been shown in Table 2. In addition, alongside those compounds, several compounds from Section 2.1. were evaluated for their antimicrobial potential. Alternarosides A–C (**4**–**6**) and alternarosin A (**7**) were identified from the fungus *Alternaria raphani* THW-18, which was obtained from sediments collected in the Hongdao sea salt field, Qingdao, China [9]. Using the agar dilution technique, these compounds produced weak antibacterial activity against *E. coli*, *B. subtilis*, and *C. albicans*, with MIC values ranging from 70 to 400 µM [9]. The compounds xanalteric acids I (**8**) and II (**9**), alternarian acid (**10**), alternarienonic acid (**94**), altenusin (**95**), altertoxin I (**52**), altenuene (**96**), 4′-*epi*-altenuene (**97**), alternariol (**41**), alternariol-5-*O*-methyl ether (**98**), alterperylenol (**68**), and stemphyperylenol (**55**) were purified and characterized from the endophytic fungus *Alternaria* sp. JCM9.2, which was isolated from *Sonneratia alba* mangrove collected in China [10]. Antibiotic activity against multi-resistant bacterial and fungal strains was evaluated for all compounds against *E. coli*, *S. aureus*, *K. pneumoniae*, *E. cloacae*, *E. faecium*, *Pseudomonas aeruginosa*, *S. pneumonia*, *A. baumanii*, *C. krusei*, *C. albicans*, *A. fumigatus*, and *Aspergillus faecalis*. In these analyses, the MIC values for xanalteric acids I (**8**) and II (**9**) against *S. aureus* ranged from 343.4 to 686.8 µM. Altenusin (**95**) showed extensive antibacterial activity against various resistant pathogens with MIC values of 107.7–431.0 µM, while all other compounds had no antibiotic action against the bacteria and fungi tested [10].

The fungus *Alternaria* sp. SF-5016, which was separated from Masan Bay shoreline sediment, provided a cyclic pentadepsipeptide, alternaramide (**99**) [33]. The antimicrobial action of compound **99** at 400 μg/disk was investigated against *S. aureus* and *Bacillus subtilis*, producing inhibition zones of 8 and 13 mm, respectively. In addition, compound **99** did not show comparable antimicrobial activity against *C. albicans*, *Filobasidiella neoformans*, or *Proteus vulgaris* [33]. The perylene derivatives, 7-*epi*-8-hydroxyaltertoxin I (**53**) stemphyperylenol (**55**) and 6-*epi*-stemphytriol (**56**) were purified from the fungus *Alternaria alternata*, derived from the algal genus *Laurencia* sp. collected in the South China Sea on Weizhou Island [34]. Compounds **53**, **56**, and **55** were evaluated for their antibacterial and antifungal activities against *E. coli*, *S. aureus*, and *A. niger*. However, none of them exhibited discernible activity [34]. A cyclic peptide (**100**) obtained from the marine sediment-derived fungus *Alternaria* sp. SF-5016 displayed antibacterial activity against *Staphylococcus aureus* and *B. subtilis* [35]. Tetrahydroaltersolanols C-F (**14**–**17**), dihydroaltersolanol A (**18**), and alterporriols N, O, P, Q, and R (**24**, **25**, **13**, **101**, and **102**), in addition to seven analogs including tetrahydroaltersolanol B (**19**), altersolanol B (**20**), altersolanol C (**21**), altersolanol L (**22**), ampelanol (**23**), alterporriol C (**26**), and macrosporin (**38**) were isolated and identified from the culture broth and the mycelia of the fungus *Alternaria* sp. ZJ-2008003, a fungus from a *Sarcophyton* sp. soft coral, which was collected from the South China Sea [14]. The antibacterial activity of these compounds was evaluated against seven pathogenic bacteria (*E. coli*, *S. aureus*, *S. albus*, *B. subtilis*, *B. cereus*, *Micrococcus tetragenus*, and *Micrococcus luteus*) and two marine pathogenic bacteria (*V. anguillarum* and *V. parahemolyticus*); only altersolanol C (**21)**, alterporriol C (**26**), and macrosporin (**38**) showed strong antibacterial activity against *E. coli* and *V. parahemolyticus*, with MIC values between 0.6 and 2.5 μM. Antiviral activity against porcine reproductive and respiratory syndrome virus (PRRSV) was investigated. Tetrahydroaltersolanol C (**14**), alterporriol C (**26**), and alterporriol Q (**101**) showed IC_50_ values of 65, 39, and 22 μM, respectively [14].

The compounds pyrophen (**103**), rubrofusarin B (**104**), fonsecin (**105**), and fonsecin B (**106**), together with dimers of naphtha-pyrones, aurasperone A (**107**), aurasperone B (**108**), aurasperone C (**109**), and aurasperone F (**110**), were obtained from the fungus *Alternaria alternata* strain D2006 cultures [36]. The fungus was isolated from a soft coral, *Denderonephthya hemprichi*, collected from the Red Sea, Egypt [36]. The antimicrobial activity of these compounds was assessed by the agar diffusion method against 11 microorganisms. The fungal strain’s crude extract was highly effective against bacteria and yeast. However, only three of the isolated metabolites revealed activity; pyrophen (**103**) and rubrofusarin B (**104**) exhibited significant antifungal activities with inhibition zones of 28 and 12 mm against *C. albicans*, respectively. Additionally, aurosperone A (**107**) was effective (inhibition zone = 13 mm) against the plant-pathogenic fungus *Rhizoctonia solani* [36]. Altenusin (**95**) and a dibenzofuran derivative, porric acid D (**111**), were recovered from the marine fungus *Alternaria* sp. identified from Bohai Sea, Tianjin seawater [37]. Using agar diffusion, the antimicrobial effect of the compounds against *Staphylococcus aureus* was evaluated. Compounds **95** and **111** inhibited *S. aureus* with MIC values of 86.2 and 347.2 μM, respectively [37]. Alterporriol S (**31**), an anthranoid dimer of the alterporriol class, was discovered in the mangrove plant *Excoecaria agallocha*-associated fungus *Alternaria* sp. SK11 in the South China Sea [38]. In addition, seven anthraquinone derivatives, (+)-α-*S*-alterporriol C (**112**), hydroxybostrycin (**113**), halorosellinia A (**114**), tetrahydrobostrycin (**115**), 9α-hydroxydihydrodesoxybostrycin (**116**), austrocortinin (**117**), and 6-methylquinizarin (**118**) were also identified. All the compounds were evaluated for their ability to inhibit the *Mycobacterium tuberculosis* protein tyrosine phosphatase B (MptpB), using sodium orthovanadate as a standard. The findings showed that compound **112** is a strong inhibitor of MptpB with an IC_50_ value of 8.7 μM [38]. A cyclic tetrapeptide cyclo(l-leucyl-trans-4-hydroxy-l-prolyl-d-leucyl-trans-4-hydroxy-l-proline) (**119**) was identified from the co-culture broth of two mangrove fungi *Phomopsis* sp. K38 and *Alternaria* sp. E33 from Guangdong Province, China [39]. The dilution approach revealed that compound **119** showed moderate to high inhibitory activity against four crop-threatening fungi, including *Rhizoctonia cerealis*, *Gaeumannomyces graminis*, *Fusarium graminarum*, and *Helminthosporium sativum*. The MIC values of compound **119** against *H. sativum* were comparable to those of the positive control, triadimefon [39]. Two tetracyclopeptides were extracted from the broth of two mangrove fungi, *Phomopsis* sp. K38 and *Alternaria* sp. E33: cyclo(d-Pro-l-Tyr-l-Pro-l-Tyr) (**120**) and cyclo(Gly-l-Phe-l-Pro-l-Tyr) (**121**) [40]. The dilution technique was utilized to examine antifungal activity and moderate to strong activity against *Candida albicans*, *Gaeumannomyces graminis*, *Helminthosporium sativum*, *Rhzioctonia cerealis*, and *Fusarium graminearum* was observed, comparing to the positive control. Cyclo(Gly-l-Phe-l-Pro-l-Tyr) (**121**) was more active (MIC = 53.8–538.7 µM) than cyclo(d-Pro-l-Tyr-l-Pro-l-Tyr) (**120**) (MIC = 67.3–769.2 µM) [40].

The fungus *Alternaria alternata*, was isolated from *Litophyton arboreum* soft coral collected from the coast of Egypt in the Red Sea. There are various antimicrobial properties of *Alternaria alternata* broth extract and three isolated compounds: alternariol-9-methyl ether-3-*O*-sulphate (**39**), alternariol-9-methyl ether (**40**), and alternariol (**41**) were investigated [17]. Using the agar diffusion technique, the antimicrobial effects were determined against Gram-positive bacteria *Bacillus megaterium*, *Bacillus cereus*, *Bacillus subtilis*, and *Staphylococcus aureus*; Gram-negative bacteria *Enterobacter cloacae*, *Klebsiella pneumoniae*, and *Escherichia coli*; and yeasts *Candida albicans*, *Saccharomyces cerevisiae*, and *Aspergillus niger*. The extract of *A. alternata* had moderate activity against *B. megaterium* and *E. coli* and strong activity against *B. cereus* with inhibition diameters of 20, 15, and 12 mm, respectively. Separated compounds correlated with antibacterial activities ranging from strong to moderate effects against the same pathogens at 50 μg/disk concentration. These compounds were also examined for their ability to block HCV protease NS3-NS4A, and hepatitis virus C NS3 protease inhibitor 2 was used as positive control. The IC_50_ values of alternariol-9-methyl ether (**40**) and alternariol (**41**) against HCV NS3-NS4A were 118.3, and 46.5 μM, respectively. The IC_50_ value for alternariol-9-methyl ether-3-*O*-sulphate (**39**) was 147.7 μM, making it less potent than alternariol (**41**). These findings revealed that the inhibitory effect was reduced after C-9 methylation of alternariol (**41**) [17].

The racemic compounds of cyclohexenone and cyclopentenone derivatives, namely (±)-(4R*,5S*,6S*)-3-amino-4,5,6-trihydroxy-2-methoxy-5-methyl-2-cyclohexen-1-one (**122**) and (±)-(4S*,5S*)-2,4,5-trihydroxy-3-methoxy-4-methoxycarbonyl-5-methyl-2-cyclopenten1-one (**123**), as well as fischexanthone (**124**), were obtained from the fungus *Alternaria* sp. R6, derived from the marine semi-mangrove plant *Myoporum bontioides* A collected from Guangdong Province, China [41]. The antimicrobial activity of these compounds was evaluated. Compounds **122**–**124** displayed no activities either against Gram-positive bacterium *Staphyloccocus aureus* nor against Gram-negative bacterium *Escherichia coli* with MIC value ≥ 1265.82 μM [41]. Investigation of the sediment-derived fungus *Alternaria* sp. KJ749826 in the south Atlantic ridge revealed tricycloalternarenes I (**125**) and J (**126**) [42]. The compounds were tested for their antibacterial activities against strains of *Streptococcus pyogenes*, *Bacillus subtilis*, and *Mycobacterium smegmatis*. However, no activity was detected at a concentration of 172.4–173.4 μM [42].

Two derivatives of perylenequinone—altertoxin VII (**50**) and butylxanalterate (**51**)—an altenusin derivative nordihydroaltenuene A (**127**), and two phthalide racemates—(*S*)-isoochracinate A1 (**128**) and (*R*)-isoochracinate A2 (**129**)—together with (*S*)-alternariphent A1 (**130**), (*R*)-alternariphent A2 (**131**)**,** altertoxin I (**52**), 7-*epi*-8-hydroxyaltertoxin (**53**), stemphytriol (**54**), 6-*epi*-stemphytriol (**56**), stemphyperylenol (**55**), (*R*)-1,6-dihydroxy-8-methoxy-3a-methyl-3,3a-dihydrocyclopenta[c]iso-chromene-2,5-dione (**132**), 1-deoxyrubralactone (**133**), 6-hydroxy-8-methoxy-3a-methyl-3a,9b-dihydro-3H-furo[3,2-c]isochromene-2,5-dione (**134**), altenuene (**96**), 4′-*epi*-altenuene (**97**), (−)-(2*R*,3*R*,4a*R*)-altenuene-3-acetoxyester (**135**), dihydroaltenuene A (**136**), 3-*epi*-dihydroaltenuene A (**137**), alternariol (**41**), alternariol monomethyl ether (**138**), 3′-hydroxyalternariol-5-*O*-methyl ether (**139**), altenusin (**95**), alterlactone (**140**), altenuisol (**141**), 5′-methoxy-6-methyl-biphenyl-3,4,3′-triol (**142**), and 2,5-dimethyl-7-hydroxychromone (**143**) were obtained from the fungus *Alternaria* sp. SCSIO41014, which was isolated from *Callyspongia* sp. sponge in Guangdong Province, China [22]. Antibacterial activity against *Staphylococcus aureus* was tested for all compounds using agar filter paper diffusion. Stemphytriol (**54**) and alterlactone (**140**), at 50 µg/disc, demonstrated inhibition zones with diameters of approximately 21 and 15 mm, respectively. Furthermore, the MIC value of compound **140** was 108.5 µM, and that of compound **54** was larger than 1265.8 µM, possibly due to its poor solubility. Ampicillin, with an MIC of 17.9 µM, was used as a positive control [22].

Tricycloalternarenes K (**144**) and L (**145**), two meroterpenoids, were obtained from the marine-derived fungus *Alternaria alternata* ICD5-11, collected from the marine isopod *Ligia exotica*, collected in Shandong Province, China [43]. Compounds **144** and **145** were evaluated for antibacterial activity against *Staphylococcus aureus* and *Bacillus subtilis* using disk diffusion techniques. However, no activity was reported at 20 μg/disk [43].

Phragamide A (**146**), phragamide B (**147**), altechromone A (**148**), tenuazonic acid (**89**), altenusin (**95**), alternariol (**41**), alternariol monomethyl ether (**138**), altertoxin I (**52**), altertoxin II (**88**), and alterperylenol (**68**) were purified from the fungus *Alternaria alternata* 13A, which was obtained from *Thalassia hemprichii* and *Phragmites australis* marine plants from a saline lake in the Wadi El Natrun, Egypt [44]. Only phragamide A (**146**) demonstrated potential antibacterial activity against Gram-positive strains. Phragamide B (**147**) revealed considerable effectiveness against *Candida albicans*, but low effect against bacterial pathogens. Tenuazonic acid (**89**) showed modest action against Gram-positive bacteria. Altenusin (**95**) and alternariol (**41**) exhibited comparable antibacterial efficacy against *S. aureus*, *B. subtilis*, *P. aeruginosa*, and *C. albicans*. Additionally, both alternariol monomethyl ether (**138**) and altertoxin I (**52**) demonstrated mild antibacterial effects against *S. aureus* and *C. albicans*. Altertoxin I (**52**), altertoxin II (**88**), and alterperylenol (**68**) displayed minimal antibacterial action towards Gram-positive bacteria. This result is due to the absence of synergism in the isolated compounds compared to the entire fungal extract [44].

The isolation and characterization of five polyketides alternariol (**41**)—alternariol-9-methyl ether (**40**), altertoxin I (**52**), altertoxin II (**88**), and tenuazonic acid (**89**)—from the marine endophytic fungus *Alternaria* sp. LV52, which was obtained from the Red Sea algae *Cystoseira tamariscifolia* in Egypt, was reported [29]. The antibacterial activity of the extract and corresponding compounds was tested against a panel of tested organisms. Based on paper disk analyses, both the fungal extract and tenuazonic acid (**89**) have low to moderate efficacy against various microbiological pathogens, including *Pseudomonas aeruginosa*, *Staphylococcus aureus*, *Bacillus subtilis*, *Candida albicans*, and *Saccharomyces cerevisiae* compared to gentamycin. While other compounds were ineffective against the microorganisms studied up to 25 μL/disk [29].

Alternarialone A (**149**), curvularin derivative, alternariol 4-methyl ether (**150**), and alternariol (**41**) were obtained from the crude extract of the mangrove-derived fungus *Alternaria longipes*, isolated from the branches of *Kandelia candel* at Guangxi, China [45]. Through broth microdilution assay, all compounds were assessed for their antibacterial activity against the *Helicobacter pylori* standard strain G27 and a clinically isolated BHKS159 strain. Alternariol 4-methyl ether and alternariol (**150** and **41**) exhibited antibacterial activity against *H. pylori* G27 with MIC values of 14.3 and 62 μM, respectively, and alternariol (**41**) also exhibited antibacterial activity against *H. pylori* BHKS159 with an MIC value of 62.0 μM, while the positive control metronidazole exhibited an MIC value of 11.6 μM. However, alternarialone A (**149**) demonstrated no inhibitory impact on the two *H. pylori* strains [45].

Altermodinacid A (**93**), which is an anthraquinone, was obtained from the fungus *Alternaria* sp. X112 that was isolated from a marine fish, *Gadus macrocephalus*, residing in Yangma Island, China [31]. No quorum sensing (QS)-inhibitory activity against *Chromobacterium violaceum* (MIC > 40 µg/well) by altermodinacid A (**93**) was observed. Furthermore, no antibacterial activity (MIC > 4 µg/well) was detected against the Gram-positive bacteria *Bacillus subtilis* and *Staphylococcus aureus*, as well as against the Gram-negative bacteria *Escherichia coli* and *Pseudomonas aeruginosa* [31].

Based on the results summarized in Table 2, marine-derived *Alternaria* species produce a structurally diverse array of metabolites with antibacterial, antifungal, and antiviral activities. Many metabolites with antimicrobial effects also display cytotoxicity, suggesting overlapping mechanisms of action. For instance, alternariol (**41**) and alternariol monomethyl ether (**138**), previously discussed for their cytotoxic effects, also inhibit several bacterial strains.

Among the most potent antibacterial agents are altersolanol C (**21**), with MIC values of 0.62 μM against *E. coli* and 1.25 μM against *V. parahaemolyticus*, and macrosporin (**38**), active against the same organisms with MICs of 2.3 μM and 5.0 μM, respectively [14]. Alterporriol C (**26**) exhibited comparable potency (MIC = 2.5 μM) and additional antiviral activity against the porcine reproductive and respiratory syndrome virus (PRRSV) with IC_50_ = 39 μM [14]. Other metabolites, such as alterporriol Q (**101**) and (+)-α-S-alterporriol C (112), inhibited PRRSV with IC_50_ = 22 μM and 8.7 μM, respectively [14,37], while tetrahydroaltersolanol C (**14**) also showed antiviral activity (IC_50_ = 65 μM) and broad antibacterial effects [14].

Within the antiviral spectrum, alternariol-9-methyl ether-3-*O*-sulphate (**39)**, alternariol-9-methyl ether (**40**), and alternariol (**41**) inhibited *B. cereus*, *B. megaterium*, and *E. coli*, with IC_50_ values of 147.7 μM, 118.3 μM, and 46.5 μM, respectively, against the HCV protease NS3–NS4A [17]. Moderately active metabolites include alterporriol S (**31**) (IC_50_ = 101.4 μM) [38], altenusin (**95**) (MIC = 86.2–431 μM) [18,37,44], and porric acid D (**111**) (MIC = 347.2 μM) [37]. Weakly active compounds such as stemphytriol (**54**) (MIC > 1265.8 μM) and the highly oxygenated derivatives (**122**–**124**) (MIC = 1724–1970 μM) showed limited activity [22,41]. Finally, alternariol 4-methyl ether (**150**) exhibited notable antibacterial effects against *Helicobacter pylori* G27 and BHKS159 with MIC = 14.3 μM [45].

The overall range of antimicrobial activity shows that only a small subset of marine *Alternaria* metabolites display strong potency, with low MIC values between 0.6 and 15 μM, whereas most fall within the moderate range of 80–450 μM. Although this level of activity is considered modest in early drug discovery, several metabolites may act through unique mechanisms. For instance, alternariol (**41**) and alternariol monomethyl ether (**138**) inhibit methicillin-resistant *Staphylococcus aureus* (MRSA) by disrupting bacterial cell division through topoisomerase inhibition [46], while maintaining low cytotoxicity toward mammalian cells [46]. Such features make them promising scaffolds for next-generation antibiotics targeting multidrug-resistant (MDR) pathogens [46].

Conclusively, marine-derived *Alternaria* species yield structurally diverse metabolites spanning a wide range of antimicrobial potencies. Quinone- and perylene-based scaffolds appear central to their activity, indicating clear structure–activity relationships. Compounds such as altersolanol C, macrosporin, and alterporriol C represent particularly promising antibacterial leads. Further studies should focus on testing these metabolites against MDR clinical isolates—including MRSA, vancomycin-resistant *Enterococcus* (VRE), and multidrug-resistant *P. aeruginosa*—to assess their therapeutic potential. Continued investigation into their biosynthetic pathways and molecular targets may ultimately support the development of novel antimicrobial agents from marine *Alternaria* species [14,17,18,22,37,38,41,44,45]. Compounds with proven antimicrobial activities are listed in Table 2.

**Table 2:** Reported compounds with proven antimicrobial activities.

Compound	Organism Tested	Biological Activity	Fungus Name	Host Organism	Reference
Alternarosides A–C (**4**–**6**) Alernarosin A (**7**)	*Escherichia coli*, *Bacillus subtilis*, *Candida albicans*	MIC = 70–400 µM	*Alternaria raphanin* THW-18	Sediment	[9]
Xanalteric acid I (**8**)	*E. coli*, *Klebsiella pneumoniae*, *Enterococcus faecium*, *Enterococcus cloacae*, *Staphylococcus aureus*, *Streptococcus pneumonia*, *Pseudomonas aeruginosa*, *Acinetobacter baumanii*, *Candida albicans*, *Candida krusei*, *Aspergillus faecalis*, *Aspergillus fumigatus*	MIC = 343.4 µM (*S. aureus*)	*Alternaria* sp. JCM9.2	Mangrove *Sonneratia alba*	[10]
Xanalteric acid II (**9**)		MIC = 686.8 µM (*S. aureus*)			
Altenusin (**95**)		MIC = 107.7–431.0 µM			
		Active (*S. aureus*, *B. subtilis*, *P. aeruginosa*, *C. albicans*)*;* Antibiofilm (*B. subtilis*)	*A. alternata* 13A	Marine plant *Phragmites australis* and *Thalassia hemprichii*	[44]
		MIC = 86.2 μM (*S. aureus*)	*Alternaria* sp.	Seawater	[37]
Alternaramide (**99**)	*Bacillus subtilis*, *Staphylococcus aureus*	8 and 13 mm at 400 µg/disk	*Alternaria* sp. SF-5016	Shoreline sediment	[33]
A cyclic peptide (**100**)	*Bacillus subtilis*, *Staphylococcus aureus*	Antibacterial activity	*Alternaria* sp. SF-5016	Marine deposit	[35]
Tetrahydroaltersolanol C (**14**)	*E. coli*, *S. aureus*, *S. albus*, *Bacillus subtilis*, *B. cereus*, *M. tetragenus*, *M. luteus*, *V. parahemolyticus*, *V. anguillarum* The porcine reproductive and respiratory syndrome virus (PRRSV)	IC_50_ = 65 μM (PRRSV)	*Alternaria* sp. ZJ-2008003	*Sarcophyton* sp. soft coral	[14]
Altersolanol C (**21**)		MIC = 0.62 and 1.25 μM (*E. coli*, *V. parahemolyticus*)			
Macrosporin (**38**)		MIC = 2.3 and 5.0 μM (*E. coli*, *V. parahemolyticus*)			
Alterporriol Q (**101**)		IC_50_ = 22 μM (PRRSV)			
Alterporriol C (**26**)		MIC = 2.5 and 2.5 μM (*E. coli*, *V. parahemolyticus*); IC_50_ = 39 μM (PRRSV)			
Pyrophen (**103**)	*B. subtilis*, *S. aureus*, *S. viridochromogenes*, *E. coli*, *C. albicans*, *M. miehi*, *C.vulgaris*, *C. sorokiniana*, *S. subspicatus*, *R. solani*; *P. ultimum*	28 mm at 40 μg/disk (*C. albicans*)	*Alternaria alternata* D2006	Soft coral, *Denderonep-hthya hemprichi*	[36]
Rubrofusarin B (**104**)		12 mm at 40 μg/disk (*C. albicans*)			
Aurasperone A (**107**)		13 mm at 40 μg/disk (*R. solani*)			
Porric acid D (**111**)	*Staphylococcus aureus*	MIC = 347.2 μM	*Alternaria* sp.	Seawater	[37]
Alterporriol S (**31**)	*Mycobacterium tuberculosis* protein tyrosine phosphatase B (MptpB)	IC_50_ = 101.4 µM	*Alternaria* sp. (SK11)	Mangrove *Excoecaria agallocha*	[38]
(+)-α-*S*-Alterporriol C (**112**)		IC_50_ = 8.7 µM			
cyclo(l-leucyl-trans-4-hydroxy-l-prolyl-d-leucyl-trans-4-hydroxy-l-proline) (**119**)	*G. graminis*, *R. cerealis*, *H. sativum*, *F. graminearum*	MIC = 287.6–553.0 µM	*Phomopsis* sp. K38, *Alternaria* sp. E33	Mangrove	[39]
Cyclo(d-Pro-l-Tyr-l-Pro-l-Tyr) (**120**)	*C. albicans*, *G. graminis*, *Rhzioctonia cerealis*, *H. sativum*, *F. graminearum*	MIC = 67.3–769.2 µM	*Phomopsis* sp. K38, *Alternaria* sp. E33	Mangrove	[40]
Cyclo(Gly-l-Phe-l-Pro-l-Tyr) (**121**)		MIC = 53.8–538.7 µM			
Alternariol-9-methyl ether-3-*O*-sulphate (**39**)	*B. megaterium*, *Bacillus cereus*, *B. subtilis*, *S. aureus*, *E. cloacae*, *K. pneumoniae*, *E. coli*, *C. albicans*, *S. cerevisiae*, *A. niger* HCV protease NS3-NS4A	10–17 mm at 50 μg/disk (*B. cereus*, *B. megaterium*, *E. coli);* IC_50_ = 147.7 μM (HCV NS3-NS4A)	*A. alternata*	Soft coral *Litophyton arboreum*	[17]
Alternariol-9-methyl ether (**40**)		10–15 mm at 50 μg/disk (*B. cereus*, *B. megaterium*, *E. coli*); IC_50_ = 118.3 μM (HCV NS3-NS4A)			
Alternariol (**41**)		10–14 mm at 50 μg/disk (*B. cereus*, *B. megaterium*, *E. coli*); IC_50_ = 46.5 μM (HCV NS3-NS4A)			
	MRSA (clinical)	Inhibition of MRSA DNA topoisomerase at 31.0–62.0 μM	*A. alternata*	Mangrove	[16,46]
	*S. aureus*, *B. subtilis*, *P. aeruginosa*, *C. albicans*	Active (*S. aureus*, *B. subtilis*, *P. aeruginosa*, *C. albicans*)	*A. alternata* 13A	*Phragmites australis*, *Thalassia hemprichii*	[44]
	*H. pylori* G27, BHKS159	MIC = 62.0 µM (*H. pylori* G27 and BHKS159)	*Alternaria longipes*	Mangrove, *Kandeliacandel*	[45]
(±)-(4R*,5S*,6S*)-3-Amino-4,5,6-trihydroxy-2-methoxy-5-methyl-2-cyclohexen-1-one (**122**)	*E. coli*, *S. aureus*	MIC = 1970.4 μM	*Alternaria* sp. R6	Mangrove *Myoporum bontioides*	[41]
(±)-(4S*,5S*)-2,4,5-Trihydroxy-3-methoxy-4-methoxycarbonyl-5-methyl-2-cyclopenten1-one (**123**)		MIC = 1724.1 μM			
Fischexanthone (**124**)		MIC > 1265.8 μM			
Stemphytriol (**54**)	*S. aureus*	21 mm at 50 µg/disk MIC > 1265.8 μM	*Alternaria* sp. SCSIO41014	*Callyspongia* sp. *sponge*	[22]
Alterlactone (**140**)		15 mm at 50 µg/disk MIC = 108.5 µM			
Tenuazonic acid (**89**)	*P. aeruginosa*, *S. aureus*, *B. subtilis*, *C. albicans*, *S. cerevisiae*	8–11 mm at 25 μL/disk	*Alternaria* sp. LV52	Algae, *Cystoseirata mariscifolia*	[29]
	Antimicrobial activity Gram-positive bacteria (*S. aureus*, *B. subtilis*); Gram-negative bacteria (*E. coli*, *P. aeruginosa*, *K. pneumonia*, *P. vulgaris*); yeast (*C. albicans*)	Moderate activity (Gram-positive strains); Antibiofilm; Gram-positive (70–80%); Gram-negative strains (40–60%)	*A. alternata* 13A	Marine plant *Phragmites australis* and *Thalassia hemprichii*	[44]
Phragamide A (**146**)	Antimicrobial activity, Gram-positive bacteria (*S. aureus*, *B. subtilis*); Gram-negative bacteria (*E. coli*, *P. aeruginosa*, *K. pneumonia*, *P. vulgaris*); yeast (*C. albicans*)	Moderate activity (*P. aeruginosa*, *C. albicans*, and Gram-positive strains); Antibiofilm, Gram-positive (70–80%); Gram-negative strains (40–60%)			
Phragamide B (**147**)		Moderate activity (*C. albicans)*; Antibiofilm; Gram-positive (70–80%); Gram-negative strains (40–60%)			
Altechromone A (**148**)		Antibiofilm, Gram-positive (70–80%), Gram-negative strains (40–60%)			
Alternariol monomethyl ether (**138**) Altertoxin I (**52**)		Weakly active (*S. aureus*, *C. albicans*)			
Altertoxin II (**88**) Alterperylenol (**68**)		Weakly active (Gram-positive strains)			
Alternariol 4-methyl ether (**150**)	*H. pylori* G27, BHKS159	MIC = 14.3 µM (*H. pylori* G27)	*Alternaria longipes*	Mangrove, *Kandeliacandel*	[45]
Altermodinacid A **(93)**	Quorum sensing (QS)-inhibitory activity against *C. violaceum*	MIC > 40 µg/well	*Alternaria* sp. X112	Marine fish *Gadus macroceph-alus*	[31]
	*B. subtilis*, *S. aureus*, *E. coli*, *P. aeruginosa*	MIC > 4 µg/well			

Note: Antimicrobial activities are listed as MIC or IC_50_ where available, or inhibition zone diameters for disk diffusion tests. IC_50_ and MIC values for all compounds have been converted to μM for consistency. For disk diffusion, a larger zone of inhibition = more potent. Table 1 shows each compound only once if evaluated in different screening platforms against different targets. Compounds **4**–**9** appear in this table (marked by their number)—their structures were given in Figure 1 earlier.

### 2.3. Compounds with Antiparasitic Activities (Table 3)

Compounds evaluated for their antiparasitic activity are shown in Figure 3, Figure 7, Figure 10 and Figure 11. Two dimeric compounds of the alternariol class, (±)-alternarlactones A (**150**) and B (**151**), verrulactone B (**152**), altenuisol (**141**), alternariol (**41**), 5-*O*-methyl ether-3-hydroxyalternariol (**139**), 4-methyl ether alternariol (**153**), alterlactone (**140**), altenuic acid II (**154**), altenuic acid III (**155**), 7-hydroxy-3-(2-hydroxypropyl)-5-methyl-isochromen-1-one (**156**), 5′-methoxy-6-methyl-biphenyl-3,4,3′-triol (**142**), and altenusin (**95**) were separated from the fungus *Alternaria alternata* P1210 obtained from halophyte *Salicornia* sp. roots gathered from a salt marsh near Santa Pola, Spain [47]. All isolated altenuisol derivatives were tested for their antiparasitic activities against *Trypanosoma cruzi*, *Trypanosoma brucei rhodesiense*, *Plasmodium falciparum*, and *Leishmania donovani*. All compounds, except **154**–**156**, showed inhibition towards *Trypanosoma* or *Leishmania*, indicating that antiparasitic actions require a large conjugated system with at least two aromatic rings. Monomers with a 2,3-dihydroxylphenyl group, such as alterlactone (**140**), altenuisol (**141**), 5′-methoxy-6-methyl-biphenyl-3,4,3′-triol (**142**), and altenusin (**95**) showed higher activity against *T. brucei rhodesiense* (IC_50_ < 10 µM) than compounds with a 3-hydroxylphenyl (**41** and **153**) or 3,4-dihydroxylphenyl (**139**) group. Structural dimerization, as seen in compounds **150**–**152**, limited the antiparasitic effect and led to the selective inhibition of *L. donovani* and *P. falciparum.* In contrast, altenuisol (**141**) had a basic structure and broad-spectrum antiparasitic action [47].

As shown above, several marine-derived compounds of the fungus *Alternaria* displayed exceptional antiparasitic effects against *Trypanosoma brucei rhodesiense*, *Trypanosoma cruzi*, *Leishmania donovani*, and *Plasmodium falciparum* [47]. The active metabolites belong mainly to the alternariol polyketide family, encompassing both monomeric and dimeric structures.

Altenuisol (**141**) represents one of the most potent and broad-spectrum compounds with antiparasitic effects, with an IC_50_ of 1.5 to 17.7 µM, demonstrating the strongest inhibition across multiple parasites. Likewise, verrulactone B (**152**) demonstrated strong effects on *L. donovani* and *P. falciparum*, with IC_50_ values of 2.4 and 13.5 µM, respectively. Further, (±)-alternarlactone A (**150**) and (±)-alternarlactone B (**151**) inhibited *L. donovani* with IC_50_ values of 4.7 and 8.9 µM, and *P. falciparum* with IC_50_ values of 5.9 and 9.7 µM. In addition, several of the monomeric phenolic compounds, altenusin (**95**) and 5′-methoxy-6-methyl-biphenyl-3,4,3′-triol (**142**) were highly active against *T. brucei rhodesiense*, with IC_50_ = 7.4 and 8.3 µM, respectively. Finally, IC**_50_** values of 7.2 and 11.7 µM were displayed by alterlactone (**140**) against T*. brucei rhodesiense* and *L. donovani*, respectively [47]. These results indicate that phenolic monomers containing extended conjugated systems and free hydroxyl groups represent key structural motifs for potent antiparasitic effects.

Moderately active compounds included alternariol (**41**) (IC_50_ of 15.4 µM against *L. donovani*) and 5-*O*-methyl ether-3-hydroxyalternariol (**139**) (IC_50_ of 7.5 µM against *L. donovani* and 13.6 µM against *T. brucei rhodesiense*). In contrast, 4-methyl ether alternariol (**153**) showed a reduced effect, with an IC_50_ of 31.1 µM against *P. falciparum*, emphasizing that methylation tends to reduce the antiparasitic potency. As seen in (±)-alternarlactones A and B (**150**–**151**), structural dimerization resulted in reduced selectivity in comparison to their monomeric compounds. These findings imply that the antiparasitic effect depends greatly on molecular planarity and the existence of available phenolic hydroxyl functionalities, which accelerate redox and membrane interactions with parasitic targets [47].

Some compounds, including altenuic acids II (**154**) and III (**155**) and 7-hydroxy-3-(2-hydroxypropyl)-5-methyl-isochromen-1-one (**156**), showed insignificant antiparasitic effect in the tested models, suggesting that the absence of conjugated aromatic moiety or phenolic groups is associated with reduced potency. This supports the conclusion that antiparasitic activity within *Alternaria*-derived compounds is closely coupled to their isocoumarin and phenolic core structures.

In conclusion, *Alternaria* species afford a distinct array of phenolic polyketides with contrasting degrees of antiparasitic effect. The most potent compounds, such as altenuisol (**141**), verrulactone B (**152**), (±)-alternarlactones A–B (**150**–**151**), altenusin (**95**), and alterlactone (**140**), exhibit low µM-IC_50_ values (<10 µM), rivaling known antiparasitic agents in in vitro potency. Moderately active secondary metabolites, including alternariol-type compounds, demonstrate activity in the 10–30 µM range and stay of pharmacological interest due to their broad spectrum and low cytotoxicity. The structure–activity relationships study indicate that extended aromatic conjugation and free hydroxyl functionalities are fundamental for activity, while methylation or dimerization reduces the potency. Overall, these results emphasize the potential of marine-derived *Alternaria* species as a valuable source of lead compounds for antiparasitic drug discovery, warranting further studies on mechanisms of action, selectivity, and in vivo efficacy [47]. Table 3 displays compounds with proven antiparasitic activities.

**Table 3:** Compounds with proven antiparasitic activities.

Compound	Organism Tested	Biological Activity	Fungus Name	Host Organism	Reference
(±)-Alternarlactone A (**150**)	Antiparasitic activity *T. brucei rhodesiense*, *T. cruzi*, *L. donovani*, *P. falciparum*	IC_50_ = 4.7, 5.9 µM (*L. donovani*, *P. falciparum*)	*Alternaria alternata* P1210	The halophyte *Salicornia* sp.	[47]
(±)-Alternarlactone B (**151**)		IC_50_ = 8.9, 9.7 µM (*L. donovani*, *P. falciparum*)			
Verrulactone B (**152**)		IC_50_ = 2.4, 13.5 µM (*L. donovani*, *P. falciparum*)			
Altenuisol (**141**)		IC_50_ = 1.5–17.7 µM			
Alternariol (**41**)		IC_50_ = 15.4 µM (*L. donovani*)			
5-*O*-Methyl ether-3-hydroxy- alternariol (**139**)		IC_50_ = 7.5 µM (*L. donovani*); IC_50_ = 13.6 µM (*T. brucei rhodesiense*)			
4-Methyl ether alternariol (**153**)		IC_50_ = 31.1 µM (*P. falciparum*)			
Alterlactone (**140**)		IC_50_ = 11.7 µM (*L. donovani*); IC_50_ = 7.1 µM (*T. brucei rhodesiense*)			
5′-Methoxy-6-methyl-biphenyl-3,4,3′-triol (**142**)		IC_50_ = 8.3 µM (*T. brucei rhodesiense*)			
Altenusin (**95**)		IC_50_ = 7.4 µM (*T. brucei rhodesiense*)			

### 2.4. Compounds with Antioxidant and Free Radical Scavenging Activity (Table 4)

**Figure 12:**
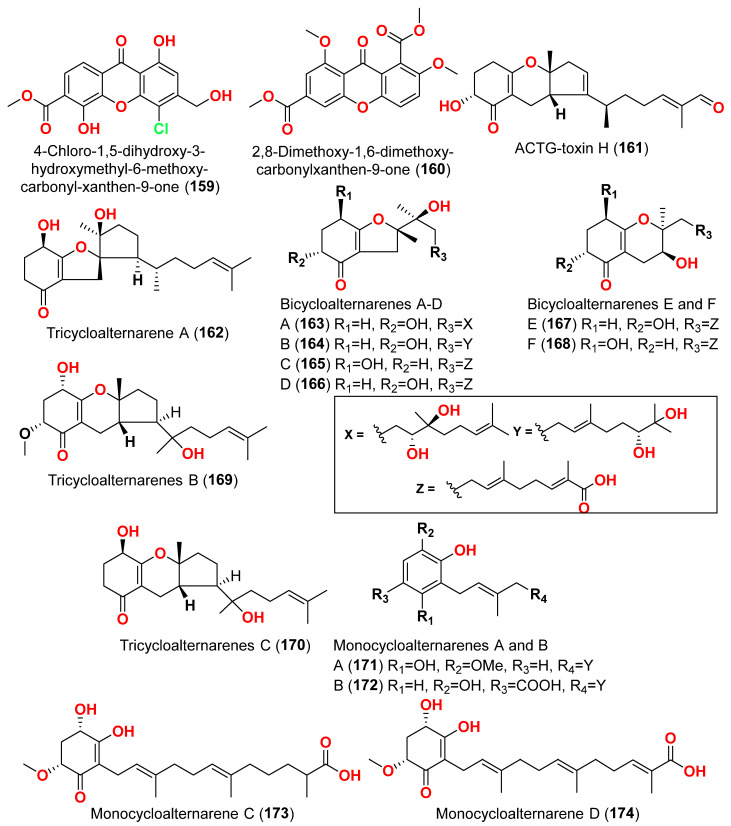
Structures of compounds **159**–**174**.

Compounds evaluated for their antioxidant and free radical scavenging effects are displayed in Figure 1, Figure 3, Figure 4, Figure 6, Figure 7, Figure 8, Figure 9, Figure 10, Figure 11 and Figure 12. Marine fungi serve as a source of natural antioxidant active substances with significant growth potential. Investigation of the fungus *Alternaria raphanin*, from sediment collected in Qingdao, China, afforded three cerebrosides, alternarosides A–C (**4**–**6**), and a diketopiperazine alkaloid, alternarosin A (**7**) [9]. The compounds were evaluated for their DPPH radical scavenging activity and did not show any activity (IC_50_ > 500 μM) [9].

The fungus *Alternaria* sp., which was isolated from a marine sponge collected in China, afforded two polysaccharides, JJY-W (**157**) and JJY-S (**158**) [48]. JJY-W (**157**) consisted mainly of galactose and glucose, along with a trace quantity of mannose. JJY-S (**158**) was mostly composed of mannose and glucose, with a trace of galactose. JJY-W (**157**) had 46% total sugar and was free of uronic acid. JJY-S (**158**) had 52% total sugar and 1.94% uronic acid. JJY-W (**157**) had a greater variety of proteins than JJY-S (**158**). The radical scavenging capabilities of compounds **157** and **158** against DPPH-free radicals and hydroxyl radicals were determined. Both compounds showed exceptional antioxidant activity. Moreover, compound **157** had a greater capacity for scavenging DPPH free radicals, while compound **158** had a greater capacity for scavenging hydroxyl radicals [48].

Three resveratrol derivatives, resveratrodehydes A–C (**44**–**46**), were isolated from the fungus *Alternaria* sp. R6, which was discovered in the mangrove plant *Myoporum bontioides* located in Guangdong Province, China [18]. Compounds **44** and **46** exhibited moderate antioxidant properties, as determined by the DPPH radical scavenging assay. The IC_50_ values of resveratrodehydes A–C (**44**–**46**) for DPPH radical scavenging activity were determined to be 447.6, >900, and 572.6 μM, respectively. These values were relatively higher compared to the IC_50_ value of the positive control resveratrol (70.2 μM) [18].

Racemic mixtures of cyclohexenone and cyclopentenone derivatives, namely (±)-(4R*,5S*,6S*)-3-amino-4,5,6-trihydroxy-2-methoxy-5-methyl-2-cyclohexen-1-one (**122**) and (±)-(4S*,5S*)-2,4,5-trihydroxy-3-methoxy-4-methoxycarbonyl-5-methyl-2-cyclopenten1-one (**123**), as well as two derivatives of xanthone, 4-chloro-1,5-dihydroxy-3-hydroxymethyl-6-methoxycarbonyl-xanthen-9-one (**159**) and 2,8-dimethoxy-1,6-dimethoxycarbonyl-xanthen-9-one (**160**), were obtained from mangrove-associated fungus *Alternaria* sp. R6, derived from marine semi-mangrove plant *Myoporum bontioides* A collected from Guangdong Province, China [41]. The scavenging activities of **122** and **123** towards ABTS [2,2′-azino-bis(3-ethylbenzthiazoline-6-sulphonic acid)] were found to be potent, with EC_50_ values of 8.1 and 16.0 μM, respectively, surpassing the activity of ascorbic acid (EC_50_ = 17.1 μM). On the other hand, compounds **159** and **160** did not exhibit any antioxidant activities (EC_50_ > 500 μM) [41].

The fungus *Alternaria* sp. SP-32 was obtained initially from a sponge collected from the South China Sea [20]. Study of the fungus revealed the extracellular polysaccharide, AS2-1 (**48**). The scavenging ability of AS2-1 (**48**) on DPPH and hydroxyl radicals was assessed and compared with those of ascorbic acid. The scavenging ability of **48** was concentration-dependent: at 36.4 µM, the effect on DPPH and hydroxyl radicals were 16.7%, and 19.2%, respectively. Similarly, the scavenging effect at a concentration of 328.4 µM was up to 90.5% on DPPH and like that of ascorbic acid on hydroxyl radicals. The EC_50_ values of **48** on DPPH and hydroxyl radicals were approximately 124 and 153.2 µM, respectively. However, less scavenging activity was observed with AS2-1 (**48**) than with ascorbic acid [20].

The fungus *Alternaria* sp. SCSIOS02F49, which was isolated from a sponge, *Callyspongia* sp., in Guangdong, China, afforded altenusin (**95**), 5′-methoxy-6-methyl-biphenyl-3,4,3′-triol (**142**), and (*S*)-alternariphent A1 (**130**) [49]. All substances were examined for their ability to scavenge DPPH free radicals. Compounds **95** and **142** exhibited significant DPPH free radical scavenging activity with IC_50_ values of 10.7 µM and 100.6 µM, respectively [49].

The fungus *Alternaria longipes*, isolated from the branches of *Kandelia candel* in Guangxi, China, provided one chromanone derivative—alterchromone A (**83**)—and four curvularin-type macrolides—curvularin (**84**), 11-β-methoxycurvularin (**85**), β,γ-dehydrocurvularin (**86**), and α,β-dehydrocurvularin (**87**) [28]. The 1,1-diphenyl-2-picrylhydrazyl (DPPH) scavenging technique was used to evaluate the antioxidant capacities of compounds **83**–**87**. Only compound **83** displayed DPPH scavenging activity, with an IC_50_ value of 160.8 μM, whereas the positive control ascorbic acid exhibited an IC_50_ of 34.0 μM [28].

Alternolides A–C (**90**–**92**), alternariol (**41**), alternariol 5-*O*-methyl ether (**98**), 3′-hydroxyalternariol-5-*O*-methyl ether (**139**), alternariol-1′-hydroxy-9-methyl ether (**215**), altenuisol (**141**), 1-deoxyrubralactone (**133**), and phialophoriol (**216**) were purified and identified from the marine-derived fungus *Alternaria alternata* LW37, which was isolated from a marine sediment [30]. The compounds were screened for their DPPH scavenging activity. Compounds **139** and **215** exhibited excellent DPPH antioxidant scavenging abilities, with IC_50_ values of 83.9 and 23.6 µM, respectively, while the positive control, ascorbic acid, had an IC_50_ value of 23.7 µM [30].

In summary, several compounds with antioxidant and radical scavenging effects have been reported from marine-derived *Alternaria*, including macrolides, phenolic polyketides, cyclohexenones, and polysaccharides. The compounds were mainly assessed through DPPH, ABTS, and hydroxyl assays, and exhibited a broad range of potency, depending on structural motifs and the degree of aromatic conjugation. The cyclohexenone derivatives (±)-(4R*,5S*,6S*)-3-amino-4,5,6-trihydroxy-2-methoxy-5-methyl-2-cyclohexen-1-one (**122**) and (±)-(4S*,5S*)-2,4,5-trihydroxy-3-methoxy-4-methoxycarbonyl-5-methyl-2-cyclopenten-1-one (**123**), which displayed strong ABTS radical scavenging with EC_50_ of 8.1 and 16.0 μM, exceeding that of ascorbic acid (EC_50_ = 17.1 μM), represent the most potent compounds [41]. Equally, altenusin (**95**) and alternariol-1′-hydroxy-9-methyl ether (**215**) displayed high DPPH radical scavenging with IC_50_ values of 10.7 and 23.6 μM, respectively, comparable to ascorbic acid (IC_50_ = 23.7 μM) [30,49]. These findings indicate that para- and ortho-hydroxylated aromatic moieties foster single-electron transfer and hydrogen atom transfer, leading to stabilizing phenoxyl radicals through conjugation and enhancing redox reactivity.

With IC_50_ values of 80 and 160 μM, alterchromone A (**83**), 3′-hydroxyalternariol-5-*O*-methyl ether (**139**), and 5′-methoxy-6-methyl-biphenyl-3,4,3′-triol (**142**) represent moderately effective compounds [28,30,49]. The polysaccharide AS2-1 (**48**) demonstrated moderate DPPH and hydroxyl radical scavenging with EC_50_ = 124 and 153 μM, while JJY-W (**157**) and JJY-S (**158**) showed complementary effects, with JJY-S more active against hydroxyl radicals and JJY-W more active against DPPH [20,48]. Their antioxidant mechanisms are likely dependent on hydrogen-donating and metal ion chelation pathways, facilitated by uronic acid and protein residues that stabilize radicals.

In contrast, weak or inactive compounds included resveratrodehydes A–C (**44**–**46**), with IC_50_ values of 447.6, >900, and 572.6 μM, respectively, and the xanthone derivatives (**159**) and (**160**), both showing EC_50_ > 500 μM [18,41]. The diminished activity of these compounds is attributed to halogen substitution or methylation, which hinders electron delocalization and lowers hydrogen-donating ability.

Mechanistic insights suggest that phenolic compounds modulate oxidative signaling and quench radicals directly. For example, alternariol (**41**) and altenusin (**95**) reduce COX-2 and iNOS by suppressing the expression of ROS-induced activation of NF-κB and Nrf2 pathways, and thereby mitigating oxidative inflammation [25,49]. These dual antioxidant and anti-inflammatory mechanisms support their role as redox-regulating agents.

Finally, compounds from *Alternaria* display a wide range of antioxidant effectiveness, from highly active, low-μM phenolics to moderately acting polysaccharides. Altenusin (**95**), alternariol-1′-hydroxy-9-methyl ether (**215**), and cyclohexenones (**122**–**123**) are the most potent candidates, competing with the activity of ascorbic acid, while hydroxyl-rich polysaccharides exhibit complementary radical scavenging through hydrogen donation and chelation. These results emphasize *Alternaria* as a vital source of both small-molecule and macromolecular antioxidant effects, through the combined mechanisms of biological redox modulation and chemical quenching [18,20,25,28,30,41,48,49]. Table 4 illustrates compounds with proven antioxidant effects.

**Table 4:** Reported compounds with antioxidants and free radical scavenging activities.

Compound	Applied Assay	Biological Activity	Fungus Name	Host Organism	Reference
JJY-W (**157**)	DPPH free radical and hydroxyl radical scavenging activity	More scavenging capacity on DPPH free radical	*Alternaria* sp.	Sponge	[48]
JJY-S (**158**)		More hydroxyl radical scavenging capacity			
Resveratrodehyde A (**44**)	DPPH scavenging activity	IC_50_ = 447.6 μM	*Alternaria* sp. R6	Mangrove *Myoporum bontioides*	[18]
Resveratrodehyde C (**46**)		IC_50_ = 572.6 μM			
(±)-(4R*,5S*,6S*)-3-Amino-4,5,6-trihydroxy-2-methoxy-5-methyl-2-cyclohexen-1-one (**122**)	ABTS radical scavenging activity	EC_50_ = 8.1 μM	*Alternaria* sp. R6	Mangrove *Myoporum bontioides*	[41]
(±)-(4S*,5S*)-2,4,5-Trihydroxy-3-methoxy-4-methoxycarbonyl-5-methyl-2-cyclopenten1-one (**123**)		EC_50_ = 16.0 μM			
AS2-1 (**48**)	DPPH and hydroxyl radical scavenging activity	EC_50_ = 124 μM (DPPH) EC_50_ = 153.2 μM (Hydroxyl radicals)	*Alternaria* sp. SP-32	Unspecified sponge	[20]
Altenusin (**95**)	DPPH scavenging activity	IC_50_ = 10.7 µM (DPPH)	*Alternaria* sp. SCSIOS02F49	Sponge, *Callyspongia* sp.	[49]
5′-Methoxy-6-methyl-biphenyl-3,4,3′-triol (**142**)		IC_50_ = 100.6 µM (DPPH)			
Alterchromone A (**83**)	DPPH scavenging activity	IC_50_ = 160.8 μM	*Alternaria longipes*	Mangrove *Kandelia candel*	[28]
3′-hydroxyalternariol-5-*O*-methyl ether (**139**)	DPPH scavenging activity	IC_50_ = 83.9 µM	*Alternaria alternata* LW37	Sediment	[30]
Alternariol-1′-hydroxy-9-methyl ether (**215**)		IC_50_ = 23.6 µM			

Note: Antioxidants and free radical scavenging activities are listed as IC_50_ or EC_50_ where available. IC_50_ and EC_50_ values for all compounds have been converted to μM for consistency.

### 2.5. Compounds with Anti-Inflammatory Activity (Table 5)

**Figure 13:**
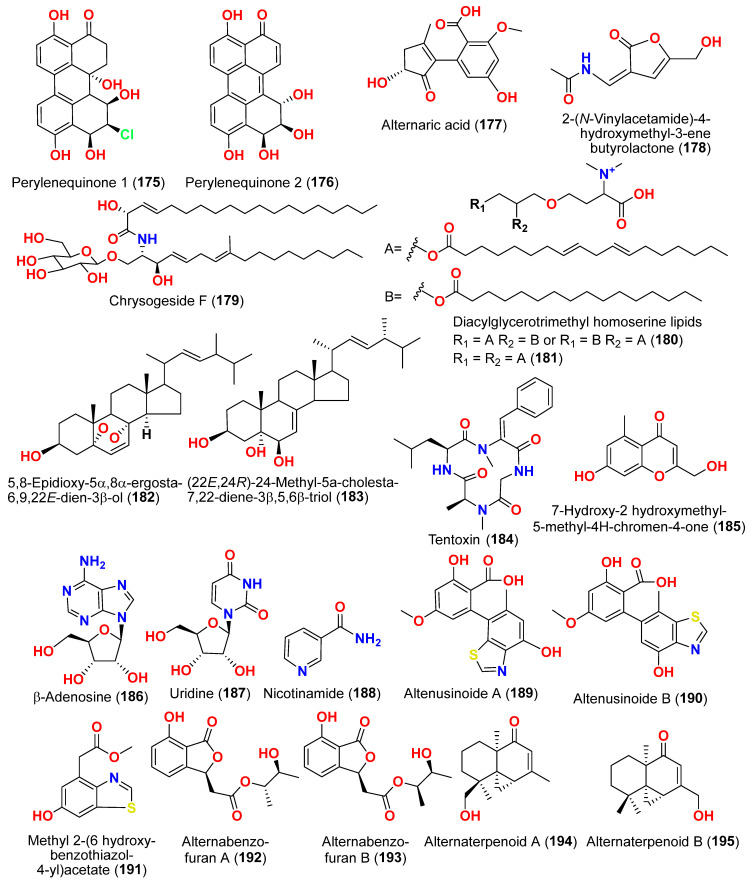
Structures of compounds **175**–**195**.

**Figure 14:**
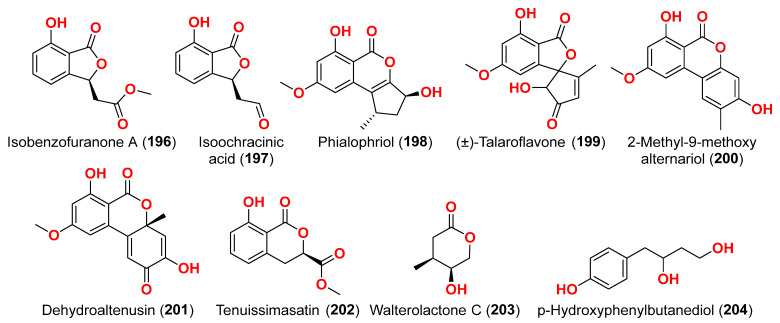
Structures of compounds **196**–**204**.

**Figure 15:**
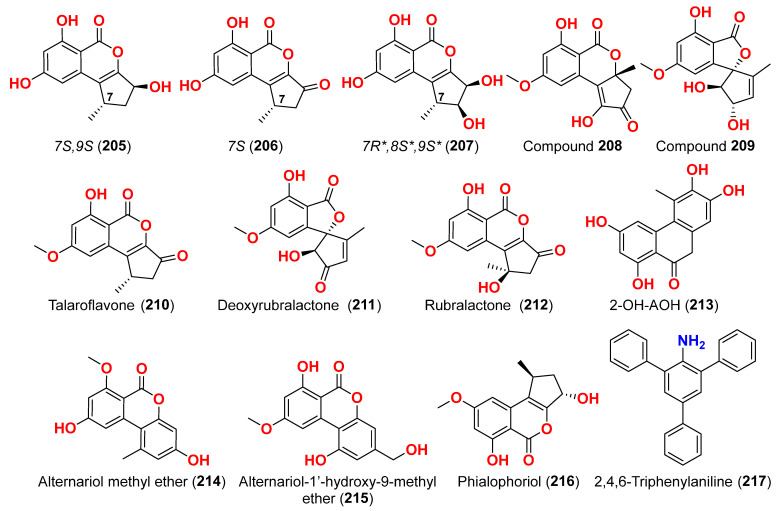
Structures of compounds **205**–**217**.

Compounds evaluated for their anti-inflammatory effects are displayed in Figure 7, Figure 9, Figure 10, Figure 12, Figure 13, Figure 14 and Figure 15. Alternaramide (**99**) was isolated from the marine-derived fungus *Alternaria* sp. SF-5016, which was obtained from a shoreline sediment sample in the Masan Bay region of Korea [33]. Compound **99** weakly inhibited protein tyrosine phosphatase 1B (PTP1B) activity by 49% at 255.1 µM [33].

Alternaramide (**99**), which was purified from the fungus *Alternaria* sp. SF-5016 extract, showed a significant decrease in LPS-stimulated RAW264.7 and BV2 cells, mRNA and protein levels of Toll-like receptor 4 (TLR4), and myeloid differentiation primary response gene 88 (MyD88) [50]. Multiple TLR4-mediated inflammatory pathways were found to be affected by alternaramide (**99**), indicating its potential to treat inflammatory and neuro-inflammatory diseases [50].

ACTG-toxin H (AH) (**161**) was obtained from a sponge-derived fungus *Alternaria alternata* sp. tzp-11, which was gathered in China [51]. The molecular mechanism underlying the anti-inflammatory properties of compound **161** was investigated. Interleukin-6, IL-1b, inducible nitric oxide synthase, cyclooxygenase-2 expression, and nitric oxide generation were reduced by compound **161** treatment in a dose-dependent manner when triggered by lipopolysaccharide (LPS). Additionally, **161** prevented the activation of P38 MAPK and Akt by LPS in RAW264.7 cells. According to electrophoretic mobility shift assays (EMSAs), compound **161** reduced the LPS-induced nuclear factor-jB (NFjB) DNA-binding activity. The transfection of toll-like receptor 4 (TLR4) increased LPS-induced NFjB transcription activity in 293T cells, determined by a transfection test and evaluation of an NFjB-sensitive promoter region. In TLR4-transfected cells, compound **161** dramatically inhibited LPS-induced NFjB activation. The anti-inflammatory properties of **161** resulted from inhibiting pro-inflammatory cytokines and enzyme production through the TLR4/NFjB signaling pathway. RAW264.7 macrophages exhibit reduced release of IL-1b, IL-6, iNOS, COX-2, and NO due to LPS-induced Akt and p38 MAPK activation [51].

The fungus *Alternaria* sp. JJY-32 was isolated from the sponge *Callyspongia* sp. collected on the coast of Hainan Island, China [52]. Investigation of the fungus revealed fifteen meroterpenoids, including tricycloalternarene A (**162**), bicycloalternarenes A–F (**163**–**168**), tricycloalternarenes B and C (**169** and **170**), ACTG-Toxins D and H (**62** and **161**), and monocycloalternarenes A–D (**171**–**174**). The NF-κB inhibitory activities in the RAW264.7 cells of these compounds were evaluated. Compounds **162**–**164** exhibited weak to moderate inhibition, with IC_50_ values ranging from 52 to 85 μM, while compounds **62**, **161**, and **167**–**172** showed IC_50_ values from 39 to 76 μM compared to the positive control PDTC, which had an IC_50_ value of 3 μM [52].

The fungus *Alternaria* sp. NH-F6 was identified from South China Sea deep sediment samples [53]. Its ethyl acetate extract produced two perylenequinones—1 and 2 (**175** and **176**)—alternaric acid (**177**), 2-(*N*-vinylacetamide)-4-hydroxymethyl-3-ene butyrolactone (**188**), and a cerebroside, chrysogeside F (**179**), together with alternarienonic acid (**94**), talaroflavone (**210**), alternariol (**41**), alternariol 5-*O*-methyl ether (**98**), 4′-epialtenuene (**97**), altenuene (**96**), diacylglycerotrimethyl homoserine lipids (**180** and **181**), 5,8-epidioxy-5α,8α-ergosta-6,22*E*-dien-3β-ol (**27**), 5,8-epidioxy-5α,8α-ergosta-6,9,22*E*-dien-3β-ol (**182**), (22*E*,24*R*)-24-methyl-5α-cholesta-7,22-diene-3β,5,6β-triol (**183**), altenusin (**95**), tentoxin (**184**), tricycloalternarene A (**162**), 2,5-dimethyl-7-hydroxychromone (**143**), 7-hydroxy-2-hydroxymethyl-5-methyl-4H-chromen-4-one (**185**), β-adenosine (**186**), uridine (**187**), and nicotinamide (**188**). All compounds were assessed for their inhibitory effect on BRD4 protein. Compound **176** exhibited a potent inhibition rate of 88.1%. Compound **185** had a modest inhibition rate of 57.7%, while other compounds were below 35.0% at a concentration of 10 µM [53].

The fungus *Alternaria* sp. SCSIOS02F49—isolated from a sponge, *Callyspongia* sp., in Guangdong, China—afforded two altenusin derivatives and thiazole hybrids—altenusinoide A (**189**), altenusinoide B (**190**), and methyl 2-(6-hydroxybenzothiazol-4-yl)acetate (**191**)—altenusin (**95**), 5′-methoxy-6-methyl-biphenyl-3,4,3′-triol (**142**), and (*S*)-alternariphent A1 (**130**) [49]. All substances were examined for their ability to inhibit COX-2. Compound **142** showed COX-2 inhibitory activity with an IC_50_ value of 9.5 µM [49].

The marine-derived fungus *Alternaria* sp. 5102, was isolated from an Actiniae (sea anemone) collected on the Laishizhou island, Guangdong Province, China [54]. Alternabenzofurans A and B (**192** and **193**), alternaterpenoids A and B (**194** and **195**), together with the polyketides, isobenzofuranone A (**196**), isoochracinic acid (**197**), (*R*)-1,6-dihydroxy-8-methoxy-3a-methyl-3,3a-dihydrocyclopenta[c]isochromene-2,5-dione (**132**), dihydroaltenuene A (**136**), phialophriol (**198**), (±)-talaroflavone (**199**), alternariol-9-*O*-methyl ether (**40**), alternariol (**41**), 2-methyl-9-methoxy alternariol (**200**), 3′-hydroxyalternariol 5-*O*-methyl ether (**139**), alternariol-1′-hydroxy-9-methyl ether (**215**), dehydroaltenusin (**201**), alteryulactone (**140**), tenuissimasatin (**202**), 5′-methoxy-6-methyl-biphenyl-3,4,3′-triol (**142**), altenusin (**95**), 2,5-dimethyl-7-hydroxychromone (**143**), and walterolactone C (**203**) were purified and identified from the extract of this fungus [52]. The compounds were tested for their ability to reduce the production of nitric oxide (NO) in RAW264.7 cells stimulated with lipopolysaccharide (LPS). Indomethacin was used as a positive control with an IC_50_ of 35.8 µM in the Griess assay. Fourteen compounds showed higher anti-inflammatory efficacy than indomethacin. Among them, compounds **194**, **132**, **198**, and **139** exhibited considerable inhibition of nitric oxide (NO) generation, with IC_50_ values below 10 µM (ranging from 1.3 to 5.9 µM). Compounds **196** and **215** exhibited moderate anti-inflammatory activities with IC_50_ values of 41.1 and 39.0 µM, respectively [54]. The marine-derived fungus *Alternaria alternata* 114-1G afforded *p*-hydroxyphenylbutanediol (**204**) with an anti-inflammatory effect [55].

As shown from the discussion above, different marine-derived *Alternaria* species afforded several compounds with prominent anti-inflammatory effects, mainly through the inhibition of pro-inflammatory mediators, including cytokines (TNF-α, IL-6), nitric oxide (NO), and enzymes including iNOS and COX-2, in LPS-stimulated microglial (BV2) cells or macrophages (RAW264.7). The NF-κB and TLR4 signaling pathways include other targets, suggesting diverse mechanisms of immunomodulation. Phialophriol (**198**) presented as the most potent compound, with an IC_50_ value of 1.3 μM, followed by alternaterpenoid A (**194**), (*R*)-1,6-dihydroxy-8-methoxy-3a-methyl-3,3a-dihydrocyclopenta[c]isochromene-2,5-dione (**132**), and 3′-hydroxyalternariol 5-*O*-methyl ether (**139**), with IC_50_ values of 2.4, 5.2, and 5.9 μM, respectively [49,54]. Further, as a potent COX-2 inhibitor, 5′-methoxy-6-methyl-biphenyl-3,4,3′-triol (**142**) showed an IC_50_ value of 9.5 μM [49]. These values highlight the pharmacological promise of these *Alternaria*-derived metabolites as anti-inflammatory leads.

Several compounds displayed moderate anti-inflammatory activity in cell-based assays, with IC_50_ values ranging from 14.9 to 26.3 μM, including walterolactone C (**203**), alteryulactone (**140**), alternariol (**41**), 2,5-dimethyl-7-hydroxychromone (**143**), alternabenzofuran B (**193**), (±)-talaroflavone (**199**), tenuissimasatin (**202**), 2-methyl-9-methoxyalternariol (**200**), altenusin (**95**), and alternariol-1′-hydroxy-9-methyl ether (**215**), with IC_50_ values of 14.9, 16.2, 16.6, 17.3, 18.7, 23.9, 24.5, 25.4, and 26.3 μM, respectively [49,54,55]. Also, ACTG-toxin D and H (**62**, **161**), tricycloalternarenes A, B, and C (**162**, **169**, **170**), bicycloalternarenes A–D (**163**–**166**), and monocycloalternarenes A–D (**171**–**174**) inhibited NF-κB activation in LPS-stimulated RAW264.7 macrophages, with IC_50_ values ranging from 39 to 85 μM [52], indicating moderate but biologically relevant effects.

Alternaramide (**99**), which inhibited PTP1B by 49% at 255.1 μM, represents example of weakly active candidate [33].

Mechanistic studies suggest that many of these compounds achieve their anti-inflammatory effect by affecting the NF-κB signaling cascade, thereby lowering the downstream expression of COX-2 and iNOS. For example, COX-2 and iNOS expression in LPS-stimulated macrophages by blocking NF-κB nuclear translocation was significantly suppressed by alternariol (**41**) [25]. Similarly, the inhibition of TLR4/NF-κB activation in microglial and macrophage cells by ACTG-toxins D and H (**62** and **161**) resulted in the reduced production of TNF-α and IL-1β [51]. These results indicate that perylenequinone-type and chromone-based compounds could serve as scaffolds for the development of anti-inflammatory or neuroprotective leads.

In conclusion, the anti-inflammatory effects of marine-*Alternaria* compounds reach a wide range of potencies. Compounds such as phialophriol (**198**), alternaterpenoid A (**194**), and hydroxylated isocoumarins display strong inhibition at sub-10 μM concentrations, exceeding the potency of standard drugs. Compounds with moderate effect, such as alternariol derivatives, present consistent μM inhibition of NO or cytokine production and are biologically relevant. Most of them act by suppressing the COX-2/iNOS or NF-κB pathways, underscoring their therapeutic potential. Further investigations into their selectivity, cytotoxicity, and in vivo anti-inflammatory efficacy are warranted to validate their potential as lead molecules for anti-inflammatory drug discovery [32,49,51,52,53,54,55]. Table 5 displays reported compounds with proven anti-inflammatory activities.

**Table 5:** Reported compounds with anti-inflammatory activities.

Compound	Applied Assay	Biological Activity	Fungus Name	Host Organism	Reference
Alternaramide (**99**)	Inhibition of protein tyrosine phosphatase 1B (PTP1B)	49% inhibition at 255.1 µM	*Alternaria* sp. SF-5016	Shoreline sediment sample	[32]
	Anti-inflammatory effect in LPS-stimulated RAW264.7 and BV2 cells	Inhibits LPS-stimulated expression of TLR4 and MyD88			[50]
ACTG-toxin H (AH) (**161**)	Pro-inflammatory cytokines and enzyme production by the TLR4/NFjB signaling pathway	Significantly inhibits LPS-induced NFjB activation in TLR4-transfected cells	*Alternaria* sp. tzp-11	Sponge	[51]
Tricycloalternarene A (**162**) Bicycloalternarenes A–D (**163**–**166**)	NF-κB inhibitory activities in RAW264.7 cells	IC_50_ = 52–85 μM	*Alternaria* sp. JJY-32	Sponge *Callyspongia* sp.	[52]
Tricycloalternarenes B, C (**169**, **170**) ACTG-toxin D, H (**62**, **161**) Monocycloalternarenes A–D (**171**–**174**)		IC_50_ = 39–76 μM			
Perylenequinone 1 (**175**)	Inhibition of BRD4 protein	57.7% inhibition rate	*Alternaria* sp. NH-F6	Deep-sea sediment	[53]
Perylenequinone 2 (**176**)		88.1% inhibition rate			
Alternaric acid (**177**); 2-(*N*-Vinylacetamide)-4-hydroxymethyl-3-ene -butyrolactone (**188**); Chrysogeside F (**179**); Alternarienonic acid (**94**); Talaroflavone (**210**); Alternariol (**41**); Alternariol 5-*O*-methyl ether (**98**); 4′-Epialtenuene (**97**); Altenuene (**96**); Diacylglycerotrimethyl Homoserine lipids (**180**, **181**); 5,8-Epidioxy-5α,8α-ergosta-6,22*E*-dien-3β-ol (**27**); 5,8-Epidioxy-5α,8α-ergosta-6,9,22*E*-dien-3β-ol (**182**); (22*E*,24*R*)-24-Methyl-5α-cholesta-7,22-diene-3β,5,6β-triol (**183**); Altenusin (**95**); Tentoxin (**184**); Tricycloalternarene A (**162**); 2,5-Dimethyl-7-hydroxychromone (**143**); 7-Hydroxy-2-hydroxymethyl-5-methyl-4H-chromen-4-one (**185**); β-Adenosine (**186**); Uridine (**187**); Nicotinamide (**188**)		<35.0% inhibition rate			
5′-Methoxy-6-methyl-biphenyl-3,4,3′-triol (**142**)	COX-2 inhibition	IC_50_ = 9.5 µM (COX-2)	*Alternaria* sp. SCSIOS02F49	Sponge, *Callyspongia* sp.	[49]
Alternabenzofuran B (**193**)	Inhibition of NO production by lipopolysaccharide (LPS) activated in RAW264.7 cells	IC_50_ = 18.7 µM	*Alternaria* sp. 5102	Actiniae	[54]
Alternaterpenoid A (**194**)		IC_50_ = 2.4 µM			
Isobenzofuranone A (**196**)		IC_50_ = 41.1 µM			
(*R*)-1,6-Dihydroxy-8-methoxy-3a-methyl-3,3a dihydrocyclopenta [c]isochromene-2,5-dione (**132**)		IC_50_ = 5.2 µM			
Phialophriol (**198**)		IC_50_ = 1.3 µM			
(±)-Talaroflavone (**199**)		IC_50_ = 23.9 µM			
Alternariol-9-*O*-methyl ether (**40**)		IC_50_ = 39.0 µM			
Alternariol (**41**)		IC_50_ = 16.6 µM			
2-Methyl-9-methoxy alternariol (**200**)		IC_50_ = 24.5 µM			
3′-Hydroxyalternariol 5-*O*-methyl ether (**139**)		IC_50_ = 5.9 µM			
Alternariol-1′-hydroxy-9-methyl ether (**215**)		IC_50_ = 26.3 µM			
Alteryulactone (**140**)		IC_50_ = 16.2 µM			
Tenuissimasatin (**202**)		IC_50_ = 24.5 µM			
Altenusin (**95**)		IC_50_ = 25.4 µM			
2,5-Dimethyl-7-hydroxychromone (**143**)		IC_50_ = 17.3 µM			
Walterolactone C (**203**)		IC_50_ = 14.9 µM			
*p*-Hydroxyphenylbutanediol (**204**)	Anti-inflammatory	Active	*Alternaria alternata* 114-1G		[55]

Note: Anti-inflammatory effects are listed as IC_50_ in μM or as % of inhibition of different enzymes where available. IC_50_ values for all compounds have been converted to μM for consistency.

### 2.6. Compounds with Antidiabetic Activity (Table 6)

Compounds investigated for their antidiabetic potential are displayed in Figure 2, Figure 3, Figure 6, Figure 7, Figure 13 and Figure 15. The fungus *Alternaria* sp. XZSBG-1 was isolated from the sediment of China’s Salt Lake in Bange, Tibet. Its chemical investigation afforded four anthraquinone derivatives, altersolanol O (**30**), alterporriol S (**31**), alterporriol T (**32**), and alterporriol U (**33**), as well as alterporriol E (**34**), alterporriol D (**35**), alterporriol N (**24**), alterporriol A (**36**), altersolanol C (**21**), altersolanol A (**37**), and macrosporin (**38**) [16]. The capacity of all compounds to inhibit α-glucosidase was evaluated. Alterporriol T (**32**) effectively inhibits α-glucosidase, having an IC_50_ value of 7.2 μM. The remaining compounds have no inhibitory effect on α-glucosidase [16].

The fungus *Alternaria* sp. SK6YW3L was identified from mangrove *Sonneratia caseolaris* gathered in China’s Guangxi Province [56]. The fungus yielded several compounds, which were evaluated for α-glucosidase inhibitory activity. Compounds 7*S* (**206**), 7*R*,8*S*,9*S* (**177**), and 2-hydroxyalternariol (2-OH-AOH) (**213**) displayed the strongest inhibitory action, with respective IC_50_ values of 78.2, 78.1, and 64.7 µM compared to other compounds and the positive control, acarbose (IC_50_ = 553.7 µM). Compounds 7*S*,9*S* (**205**), and rubralactone (**212**) were two-fold more active than acarbose. Nonetheless, compound **208**, talaroflavone (**210**), and alternariol (**41**) had moderate inhibitory action against α-glucosidase, with IC_50_ values of 334.4, 348.4, and 474.3 µM, respectively. Other altenusin derivatives with a 6/5/5 ring skeleton, such as compound **209** and deoxyrubralactone (**211**), exhibited poor activity (IC_50_ > 500 µM). Compared to alternariol methyl ether (**214**), which contains a methoxy group, the chelated hydroxyl group at C-3 (**213** and **41**) increased the inhibitory effect [56].

Three dibenzo-α-pyrone derivatives, alternolides A–C (**90**–**92**), and seven congeners—alternariol (**41**), alternariol 5-*O*-methyl ether (**98**), 3′-hydroxyalternariol-5-*O*-methyl ether (**139**), alternariol-1′-hydroxy-9-methyl ether (**215**), altenuisol (**141**), 1-deoxyrubralactone (**133**), and phialophoriol (**216**)—were isolated from the marine-derived fungus *Alternaria alternata* LW37, which was obtained from a marine sediment [30]. All compounds were evaluated for their α-glucosidase inhibition effect. Compounds **91**, **92**, **215**, **141**, and **133** exhibited α-glucosidase inhibitory actions with inhibition rates of 36.6, 49.2, 93.7, 37.2, and 53.9%, respectively, at a concentration of 400 µM. Compounds **91** and **92** inhibited α-glucosidase with IC_50_ values of 725.8 and 451.2 µM, respectively, while compound **215** demonstrated considerable inhibitory action with an IC_50_ value of 6.27 µM (the positive control, acarbose, had an IC_50_ value of 1.5 µM) [30].

2,4,6-Triphenylaniline (**217**) was purified from the fungus *Alternaria longipes* strain VITN14G, which was obtained from the mangrove plant *Avicennia officinalis* collected in the Chidambaram district, India [57]. The antidiabetic activity of this compound was determined through in vitro analysis of its α-amylase and α-glucosidase inhibition. In terms of α-amylase inhibition rates, there was no significant difference observed between 2,4,6-triphenylaniline (**217**) (51.5%) and acarbose (56.6%), which is the standard drug. Conversely, the α-glucosidase inhibition rate of compound **217** (78.8%) was slightly lower than that of acarbose (89.9%), but it displayed a significantly improved inhibitory activity towards α-glucosidase. 2,4,6-Triphenylaniline (**217**) exhibited potent α-amylase inhibitory activity (IC_50_ = 84.1 μM) compared to α-glucosidase inhibition (IC_50_ = 121.4 μM) and compared to α-amylase, like the standard drug acarbose [57].

As shown from the above discussion, marine-derived *Alternaria* species have delivered several chemically diverse compounds with noteworthy antidiabetic potential, largely through the inhibition of α-amylase and α-glucosidase, the carbohydrate-hydrolyzing enzymes. α-Amylase and α-glucosidase play fundamental roles in the regulation of postprandial glucose, and their inhibition signifies a proved therapeutic methodology for managing type 2 diabetes mellitus.

Alterporriol T (**32**), with an IC_50_ value of 7.2 μM against α-glucosidase, exceeds the effect of reference drug acarbose (IC_50_ = 553.7 μM) [16] and is therefore considered as a potent candidate. Similarly, the dibenzo-α-pyrone derivative alternariol-1′-hydroxy-9-methyl ether (**215**) displayed significant α-glucosidase inhibition with an IC_50_ value of 6.2 μM and with a 93.7% inhibition rate at 400 μM [30]. Both compounds are considered as potential candidates for future in vivo studies and development. 

Further, several phenolic and polyketide compounds, including 2-hydroxyalternariol (2-OH-AOH) (**213**), 7*S* (**206**), and 7R*,8S*,9S* (**207**) displayed significant α-glucosidase inhibition, with IC_50_ values of 64.7, 78.2, and 78.1 μM, respectively, are moderately active candidates and present a roughly seven-fold greater potency than acarbose [56]. Other compounds with moderate effects are rubralactone (**212**) (IC_50_ = 194.4 μM) and 7*S*,9*S* (**205**) (IC_50_ = 235.2 μM), which are two- to three-fold more active than the control drug. Additional moderately active compounds are talaroflavone (**210**) (IC_50_ = 348.4 μM) and alternariol (**41**) (IC_50_ = 474.3 μM), while alternariol methyl ether (**214**) and deoxyrubralactone (**211**) are considered weakly active or inactive compounds (IC_50_ > 500 μM) [56]. These data suggest that the presence of a free hydroxyl group at C-3 improves enzyme interaction, whereas ring fusion or methylation tends to diminish inhibitory potency.

In addition, alternolides B (**91**) and C (**92**) exhibited modest α-glucosidase inhibition with IC_50_ values of 725.8 and 451.2 μM, respectively, while altenuisol (**141**) and 1-deoxyrubralactone (**133**) showed inhibition rates of 37.2% and 53.9% at 400 μM [30].

2,4,6-Triphenylaniline (**217**) demonstrated dual enzyme inhibition, with IC_50_ values of 84.1 and 121.4 μM for α-amylase and α-glucosidase. Its α-glucosidase inhibition rate (78.8%) was only slightly lower than that of acarbose (89.9%), while α-amylase inhibition (51.5%) was nearly equivalent to the standard drug (56.6%) [57]. These results highlight the potential of non-phenolic aromatic scaffolds for multitargeted carbohydrate enzyme inhibition.

Collectively, the compounds display a wide range of α-amylase and α-glucosidase inhibition. The most active and potent candidates are alterporriol T (**32**) and alternariol-1′-hydroxy-9-methyl ether (**215**), with low micromolar IC_50_ values (6–7 μM), suggesting strong competitive inhibition equivalent to or surpassing standard inhibitors. Among the moderately active candidates are the hydroxylated anthraquinones and perylenequinones (IC_50_ = 60–250 μM), while methylated or dimeric analogs were less active. Structural analyses suggest that hydroxyl substitution and conjugated planar systems enhance enzyme binding through hydrogen bonding and π–π stacking, whereas methylation reduces affinity.

Mechanistically, several of these phenolic metabolites may exert dual antihyperglycemic effects by both antioxidant-mediated glucose regulation and enzyme inhibition, as previously observed for the polyketides [25,30,56,57]. Overall, these findings underline *Alternaria* as a hopeful source of structurally diverse enzyme inhibitors with potential for antidiabetic drug development, particularly in targeting postprandial glucose control through α-amylase and α-glucosidase modulation [16,30,56,57]. Compounds that show antidiabetic activities are shown in Table 6.

**Table 6:** Reported compounds with antidiabetic activities.

Compound	Applied Assay	Biological Activity	Fungus Name	Host Organism	Reference
Alterporriol T (**32**)	Inhibition of α-glucosidase	IC_50_ = 7.2 μM	*Alternaria* sp. XZSBG-1	Sediment	[16]
7*S*,9*S* (**205**)		IC_50_ = 235.2 μM	*Alternaria* sp. SK6YW3L	Mangrove *Sonneratiacaseolaris*	[56]
7*S* (**206**)		IC_50_ = 78.2 μM			
7R*,8S*,9S* (**207**)		IC_50_ = 78.1 μM			
Compound **208**		IC_50_ = 334.4 μM			
Compound **209**		IC_50_ > 500 μM			
Talaroflavone (**210**)		IC_50_ = 348.4 μM			
Deoxyrubralactone (**211**)		IC_50_ > 500 μM			
Rubralactone (**212**)		IC_50_ = 194.4 μM			
2-OH-AOH (**213**)		IC_50_ = 64.7 μM			
Alternariol (**41**)		IC_50_ = 474.3 μM			
Alternariol methyl ether (**214**)		IC_50_ > 500 μM			
Alternolide B (**91**)		IC_50_ = 725.8 µM α-glucosidase), 36.62% inhibition rate	*Alternaria alternata* LW37	Deep-sea sediment	[30]
Alternolide C (**92**)		IC_50_ = 451.2 µM α-glucosidase), 49.24% inhibition rate			
Alternariol-1′-hydroxy-9-methyl ether (**215**)		IC_50_ = 6.2 µM (α-glucosidase), 93.7% inhibition rate			
Altenuisol (**141**)		37.2% α-glucosidase inhibition rate			
1-Deoxyrubralactone (**133**)		53.9% α-glucosidase inhibition rate			
2,4,6-Triphenylaniline (**217**)	Inhibition of α-Amylase, α-glucosidase	IC_50_ = 84.1 μM (α-amylase), IC_50_ = 121.4 μM (α-glucosidase)	*Alternaria longipes* VITN14G	Mangrove plant *Avicennia officinalis*	[57]

Note: Antidiabetic effects are listed as IC_50_ in μM or as % of inhibition of α-amylase and α-glucosidase where available. IC_50_ values for all compounds have been converted to μM for consistency.

### 2.7. Compounds with Phytotoxic Activity (Table 7)

**Figure 16:**
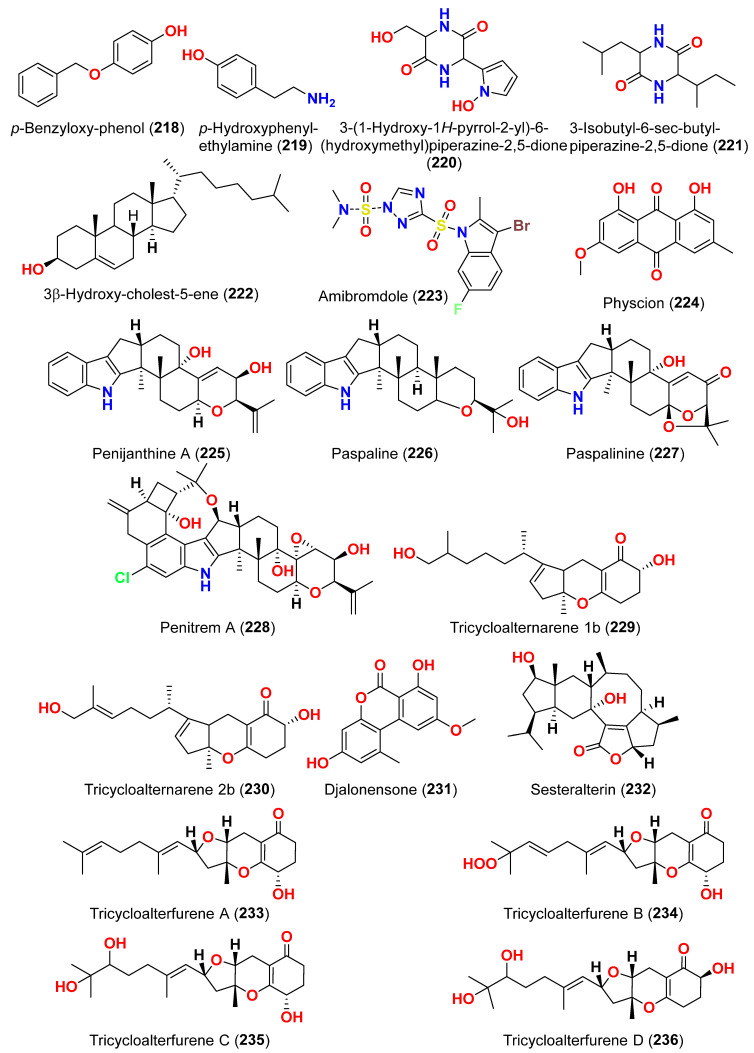
Structures of compounds **218**–**236**.

**Figure 17:**
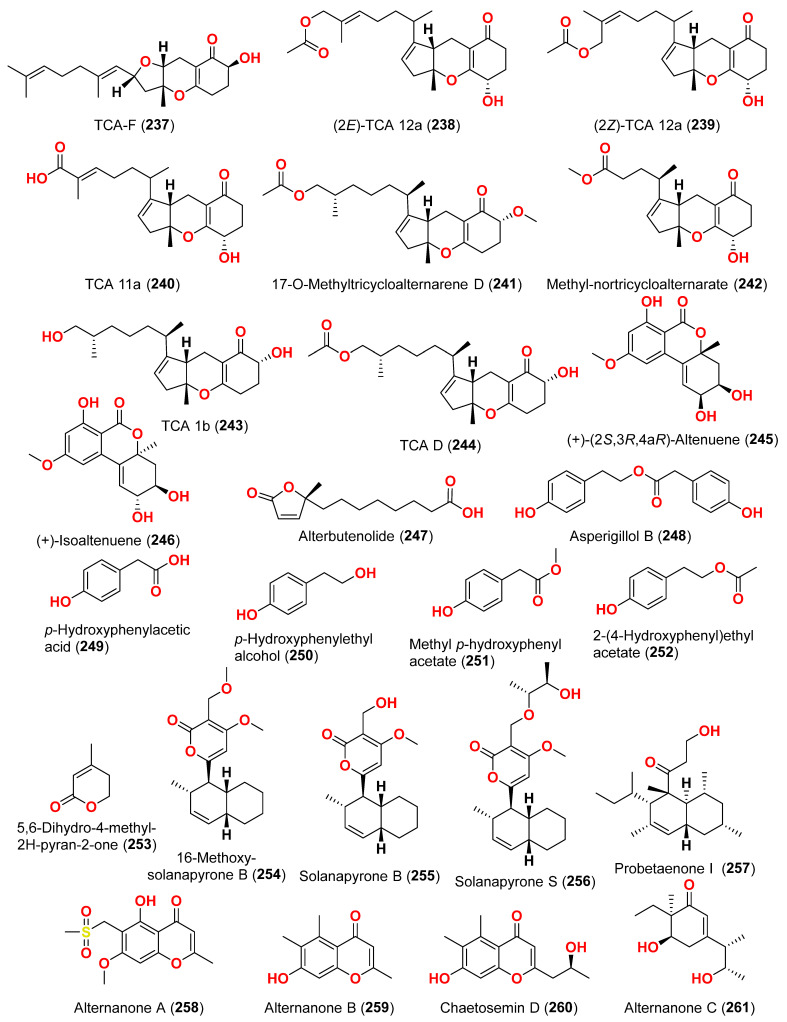
Structures of compounds **237**–**261**.

Chemical structures of the compounds evaluated for their phytotoxic activity are displayed in Figure 2, Figure 3, Figure 4, Figure 5, Figure 6, Figure 7, Figure 8, Figure 10, Figure 11, Figure 16 and Figure 17. Members of the genus *Alternaria* are among the most common plant pathogens. Their secondary metabolites have phytotoxic properties, making them valuable for agricultural applications. The compounds *p*-benzyloxy-phenol (**218**), *p*-hydroxyphenyl ethylamine (**219**), 3-hydroxymethyl-8-hydroxy-pyrrolopiperazine-2,5-dione (**220**), 3-isobutyl-6-sec-butyl-piperazine-2,5-dione (**221**), 5α,8α-epidioxy-ergosta-6,22-diene-3β-ol (**27**), and 3β-hydroxy-cholest-5-ene (**222**) were purified from the marine fungus *Alternaria* sp. [58]. Compounds **218**–**221** induced the morphological deformation of mycelia germinated from conidia of *Pyricularia oryzae* [58].

Four pyrone derivatives—pyrophen (**103**), rubrofusarin B (**104**), fonsecin (**105**), and fonsecin B (**106**)—and four dimers of naphtha-pyrones—aurasperones A–C (**107**–**109**) and F (**110**)—were reported from the fungus *Alternaria alternata* strain D2006, which was purified from the soft coral *Denderonephthya hemprichi* [36]. All compounds exhibited weak cytotoxicity (4–11% inhibition) in the brine shrimp assay at concentrations between 16.5 and 36.4 µM [36].

Four polyketides amibromdole (**223**)—altersolanol L (**22**), altersolanol C (**21**), and physcion (**224**)—were derived from the fungus *Alternaria* sp. which was isolated from a coral found in the South China Sea [59]. All polyketides were evaluated for the ability to suppress the larval settling of *B. amphitrite larvae* at concentrations between 35.9 and 176.0 μM. Moderate activity was shown by amibromdole (**223**), with an EC_50_ value of 35.9 μM [59].

The fungus *Alternaria tenuissima* EN-192 was isolated from the mangrove *Rhizophora stylosa* on Hainan Island in the South China Sea [60]. Investigation of the fungus afforded the following: four indole-diterpenoids—penijanthine A (**225**), paspaline (**226**), paspalinine (**227**), and penitrem A (**228**); three tricycloalternarene derivatives—tricycloalternarene 3a (**59**), tricycloalternarene 1b (**229**), and tricycloalternarene 2b (**230**); and two alternariol derivatives—djalonensone (**231**) and alternariol (**41**) [60]. The inhibitory activities of each isolated compound against the pathogenic bacterium *Vibrio anguillarum* were evaluated. Using the disk diffusion method, compounds **59** and **231** showed moderate activity against *V. anguillarum*, with inhibition zone diameters of 8 and 9 mm, respectively, at 100 μg/disk. In comparison, the positive control, chloramphenicol, demonstrated a significantly larger inhibition zone of 22 mm when used at a concentration of 20 µg/disk [60].

The marine-derived fungus *Alternaria* sp. WZL003, obtained from a gorgonian *Echinogorgia rebekka* collected from the South China Sea, was investigated [61]. Investigation of the fungal extract afforded macrosporin (**38**). Investigation of the antimicrobial mechanism against the marine pathogenic *Vibrio anguillarum* revealed that compound **38** effectively eradicates bacteria by causing destruction to both the cell wall and cytoplasmic membrane. This process causes an increase in cell permeability, causing the release of cellular content [61].

Alterbrasone (**49**) was isolated from the crinoid-associated fungus *Alternaria brassicae* 93, named as *Comanthina schlegeli*, collected from the South China Sea [21]. Compound **49** was evaluated against twelve aquatic bacteria: *E. coli*, *Shigella castellani*, *Salmonella*, *S. aureus*, *Vibrio parahemolyticus*, *Vibrio vulnificus*, *Vibrio alginolyticus*, *Vibrio cholera*, *Citrobacter freundii*, *Exiguobacterium aurantiacum*, *Morganella morganii*, and *Bacillus cereus*. No antibacterial activity was observed for compound **49** against all aquatic bacteria, with IC_50_ values below 60 μM [21].

Racemic mixtures of cyclohexenone and cyclopentenone derivatives, namely (±)-(4R*,5S*,6S*)-3-amino-4,5,6-trihydroxy-2-methoxy-5-methyl-2-cyclohexen-1-one (**122**), (±)-(4S*,5S*)-2,4,5-trihydroxy-3-methoxy-4-methoxycarbonyl-5-methyl-2-cyclopenten1-one (**123**), and fischexanthone (**124**), along with 4-chloro-1,5-dihydroxy-3-hydroxymethyl-6-methoxycarbonyl-xanthen-9-one (**159**) were obtained from the fungus *Alternaria* sp. R6, derived from a marine semi-mangrove plant *Myoporum bontioides* A collected from Guangdong Province, China [41]. The antimicrobial activity of these compounds was evaluated. The activities of compounds **123**, **124**, and **159** were higher than the positive control triadimefon (MIC = 510.6 μM) against *Fusarium graminearum*, with MIC values of 215.5, 474.6, and 107.1 μM, respectively. Compound **159** also exhibited more potent antifungal activity against *Calletotrichum musae* (MIC = 214.2 μM) than triadimefon (MIC = 340.4 μM), while compounds **123** and **124** showed moderate activities, with MIC values of 862.0 and 474.6 μM, respectively. Compound **122** was inactive towards *Calletotrichum musae* (MIC > 1970.4 μM). Compound **159** bears a chlorine group at C-4 and exhibits a greater antifungal activity against *Fusarium graminearum* and *Calletotrichum musae* than compound **124**, which lacks the chlorine substitution at C-4, suggesting that the chlorine group at C-4 is the key reason for better antifungal activity [41].

Sesteralterin (**232**), tricycloalterfurenes A–D (**233**–**236**), and TCA-F (**237**) were obtained from the fungal strain *Alternaria alternata* k21-1, which was isolated from the marine red alga *Lomentaria hakodatensis* collected from Kongdong Island [62]. Compounds **232**–**237** were examined for growth suppression against three marine phytoplanktons—*Chattonella marina*, *Heterosigma akashiwo*, and *Prorocentrum donghaiense*—one marine zooplankton—*Artemia salina*—and one marine-derived bacterium—*Pseudoalteromonas citrea*—that can cause porphyrayezoensis green-spot disease. Among the marine plankton tested, *C. marina* appeared more sensitive to compounds **232**–**237**, with weak to moderate inhibition at a concentration of 264.5 μM. Compound **232** showed 41–69% inhibition of the three phytoplankton, but was inactive with the zooplankton *A. salina*. Compound **233** (64, 37, 46% inhibition) and compound **237** (70, 41, 52% inhibition) showed almost similar effects against the three phytoplanktons, respectively, indicating that the hydroxyl group’s position on ring C had minimal impact on their activities. Hydroxylation at C-2 and C-3 slightly reduced the inhibition of the three phytoplanktons by compounds **235** and **236**, which showed inhibition ranges from 17 to 56%. In addition, all compounds did not exhibit activities against the *bacterium P. citrea* at 20 μg/disk [62].

Three isomeric tricycloalternaren—(2*E*)-TCA 12a (**238**), (2*Z*)-TCA 12a (**239**), and TCA 11a (**240**)—were obtained from the fungus *Alternaria alternata* k23-3 isolated from the marine red alga *Gelidiella acerosa* collected from Kongtong Island, China [63]. Compounds **238**–**240** showed weak to moderate activity by inhibiting or killing the marine plankton tested, *Chattonella marina*, *Heterosigma akashiwo*, and *Prorocentrum donghaiense*. However, compound **239** had a greater ability to suppress the three phytoplankton species and was less toxic to the zooplankton *Artemia salina* than compound **238** [63].

Two tricycloalternarene-type meroterpenes, 17-*O*-methyltricycloalternarene D (**241**) and methyl-nortricycloalternarate (**242**), and two congeners, TCA 1b (**243**) and TCA D (**244**), were purified from the extract of the fungus *Alternaria alternata* k21-1, isolated from the marine red alga *Lomentariah akodatensis* collected from Kongdong Island [64]. Evaluation of these compounds for the growth inhibition of three marine planktons—*Chattonella marina*, *Heterosigma akashiwo*, and *Prorocentrum donghaiense*—and toxicity to one marine zooplankton—*Artemia salina*—revealed weak or moderate inhibition. Among the phytoplankton species tested, *C. marina* appeared to be more sensitive to compounds **241**–**244** than *H. akashiwo* and *P. donghaiense* were, and than to compound **244** with an inhibitory rate of 78.5% at 256.4 μM. In addition, compound **244** was less toxic to the zooplankton *A. salina* than the others, possibly due to its acetyl and hydroxy groups located at C-1 and C-17, respectively [64].

The fungus *Alternaria* sp. P8 was isolated from several unidentified marine plants collected from the Yellow Sea in Qingdao, China [65]. The fungus afforded two perylenequinones, stemphyperylenol (**55**) and alterperylenol (**68**) [63]. In the antifungal assay, six plant pathogenic fungal strains, *A. alternata (Fries) Keissler*, *A. brassicicola*, *P. parasitica var. nicotianae Tucker*, *D. medusaea Nitschke*, *A. niger van. Tiegh*, and *P. theae*, were used. Stemphyperylenol (**55**) showed inhibitory activity against *P. theae* and *A. brassicicola*, with MIC values of 22.1 and 355.1 µM, respectively, which were like the positive control carbendazim. Alterperylenol (**68**) showed significant antibacterial activity against *C. michiganensis*, with an MIC of 5.57 µM, which was two times higher than the positive control of streptomycin (MIC = 6.7 µM) [65].

The marine-derived fungus *Alternaria* sp. P8 was isolated from seawater, sediments, animals, and algae collected from the Yellow Sea, South China Sea, and Chinese coastal marine environments [66]. Chemical analysis of the fungal extract resulted in the discovery of one benzopyranone, (+)-(2*S*,3*R*,4a*R*)-altenuene (**245**), and seven compounds, (+)-isoaltenuene (**246**), alternariol (**41**), altenuisol (**141**), alternariol-9-methyl ether (**40**), altertoxin I (**52**), stemphyperylenol (**55**), and alterperylenol (**68**). Compounds **245**, **246**, **52**, **55**, and **68** showed obvious phytotoxicity against the seedling growth of amaranth and lettuce at 200 ppm. The perylenequinones (**52**, **55**, and **68**) were more active than the benzopyranones (**245** and **246**), since they were able to block seed germination at 200 ppm and still exhibited high phytotoxicity at 50 ppm. It showed that separated chemicals hindered lettuce seedling development less than amaranth seedling growth. Their effects on root elongation were much more pronounced than on hypocotyl elongation. Due to the lack of observed phytotoxicity in compounds **40**, **41**, and **141**, it is revealed that substituting benzene with cyclohexene could favorably affect phytotoxicity. In addition, anti-phytopathogenic properties for compound **245** displayed strong antifungal activity against *A. brassicicola*, with an MIC of 428.0 μM, which was equivalent to that of the positive control carbendazim. Compound **52** exhibited moderate antifungal activity toward *D. medusaea*, with an MIC of 177.5 μM, compared to 163.8 μM for carbendazim. None of the compounds showed antibacterial activity [66].

A derivative of butenolide, known as alterbutenolide (**247**), containing a long-chain aliphatic acid, was discovered together with seven phenolic compounds, namely alternariol (**41**), asperigillol B (**248**), *p*-hydroxyphenylacetic acid (**249**), *p*-hydroxyphenylethyl alcohol (**250**), methyl *p*-hydroxyphenyl acetate (**251**), 2-(4-hydroxyphenyl) ethyl acetate (**252**), and 5,6-dihydro-4-methyl-2H-pyran-2-one (**253**), from the sponge-derived fungus *Alternaria alternata* I-YLW6-1 gathered from the Shandong Province of China [67]. All compounds underwent growth inhibition tests against three marine phytoplanktons—*Chattonellamarina*, *Heterosigma akashiwo*, and *Prorocentrum donghaiense*—as well as one marine zooplankton—*Artemia salina*. Only compound **247** demonstrated moderate inhibition against *C. marina*, with an IC_50_ value of 144.1 μM. On the other hand, compounds **41** and **248** exhibited significant to moderate inhibitory activities against *C. marina*, *H. akashiwo*, and *P. donghaiense*, with IC_50_ values ranging from 11.6 to 140.3 μM. Additionally, compounds **248** and **249** showed weak toxicity against brine shrimp larvae *A. salina*, with LC_50_ values exceeding 367.6 and 657.8 μM, respectively [67].

The fungus *Alternaria iridiaustralis*, derived from the *Suaeda glauca* marine plant found in the Yellow River Delta in Dongying, China, produced 16-methoxy solanapyrone B (**254**), as well as solanapyrones B and S (**255** and **256**), probetaenone I (**257**), alternanones A and B (**258** and **259**), chaetosemin D (**260**), alternanone C (**261**), and tenuazonic acid (**89**) [68]. These compounds have shown potential as herbicides, specifically in inhibiting the growth of *Echinochloa crusgalli* seedlings. Of particular significance is compound **89**, which exhibited inhibition rates exceeding 90% at concentrations of 101.5 and 203.0 µM, surpassing the effectiveness of the commonly used chemical herbicide acetochlor. Compound **259** also showed moderate inhibition rates of 60.3% and 72.6%, at of 98.0 and 196.0 µM, respectively. Additionally, compounds **258**, **259**, and **261** demonstrated antifungal properties against two carbendazim-resistant strains of *B. cinerea*, with MIC values ranging from 141.5 to 313.7 µM, which were significantly superior to those of carbendazim (MIC = 1339 µM). Furthermore, compounds **259**–**261** also displayed moderate antifungal activities against two *F. oxysporum* strains [68].

Altermodinacid A (**93**), an anthraquinone, was initially isolated from the fungal extract of *Alternaria* sp. X112, which was obtained from a marine fish *Gadus macrocephalus* found in the vicinity of Yangma Island, China [31]. However, the compound did not exhibit any significant antifungal activity against various agricultural pathogenic fungi, including *Alternaria solani*, *Lasiodiplodia pseudotheobromae*, *Fusarium oxysporum f.* sp. *cubense*, *Fusarium oxysporum f.* sp*. phaseoli*, *Fusarium foetens*, *Fusarium graminearum*, *Nectria* sp., *Fusarium mangiferae*, *Colletotrichum asianum*, *Colletotrichum musae*, and *Colletotrichum coccodesand*, as the MIC was found to be greater than 40 µg/well [31].

As shown above, marine-derived *Alternaria* species have emerged as prolific sources of chemically diverse secondary metabolites with broad-spectrum phytotoxic, and algicidal activities (Table 7).

Highly potent compounds are represented by alterperylenol (**68**) and stemphyperylenol (**55**), with complete inhibition of the seed germination of amaranth and lettuce at 50–200 ppm and antimicrobial activities surpassing streptomycin with MIC values of 5.57 µM against *Clavibacter michiganensis* and 22.1 µM against *P. theae* [65,66]. Similarly, altertoxin I (**52**) showed high phytotoxicity comparable to carbendazim at 200 ppm [66]. Exceeding the effect of the commercial herbicide acetochlor, tenuazonic acid (**89**) inhibited the growth of *Echinochloa crusgalli* at 101.5–203 µM by >90% [68]. Mechanistic studies showed that **89** acts through the inhibition of electron transport in photosystem II, leading to the accumulation of reactive oxygen species (ROS), chlorophyll degradation, lipid peroxidation, and necrosis. Similarly, macrosporin (**38**) eradicates *Vibrio anguillarum* by disrupting cytoplasmic membranes and cell walls [61]. Also, the chlorinated xanthone derivative **159** displayed superior antifungal effect against *Fusarium graminearum* and *Calletotrichum musae* (MIC 107.1–214.2 µM) relative to the reference fungicide triadimefon [41].

Amibromdole (**223**) displayed moderate antifouling activity with an EC_50_ of 35.9 µM, and altersolanol L (**22**), altersolanol C (**21**), and physcion (**224**) are example of compounds with moderate activity (EC_50_ 78.1–176 µM) [59]. Alterbutenolide (**247**) and asperigillol B (**248**) also displayed moderate algicidal effects, with IC_50_ values between 11.6 and 144.1 µM and low toxicity to zooplankton [67]. The benzopyranone derivatives altenuene (**245**) and isoaltenuene (**246**) showed moderate phytotoxicity against amaranth and lettuce at 200 ppm, while alternanones (**258**–**261**) showed moderate antifungal and herbicidal effects, including 60–72% inhibition of *E. crusgalli* and MICs of 141.5–313.7 µM against *B. cinerea* and *Fusarium oxysporum* [66,68].

Conclusively, perylenequinones and tenuazonic acid appear as the most potent candidates for bioherbicidal and antifungal applications due to their potent activity and well-understood ROS-mediated mechanisms. Moderately active scaffolds, including benzopyranones, tricycloalternarenes, and alternanones, represent potential candidates for further chemical derivatization and optimization. Cooperatively, these compounds underscore the ecological, agricultural, and biotechnological relevance of members of *Alternaria* as a source of bioactive compounds for sustainable crop protection. Table 7 displays compounds with reported phytotoxic effects.

**Table 7:** Reported compounds with proven phytotoxic activities.

Compound	Organism Used	Biological Activity	Fungus Name	Host Organism	Reference
*p*-Benzyloxy-phenol (**218**) *p*-Hydroxyphenyl ethylamine (**219**) 3-Hydroxymethyl-8-hydroxy-pyrrolopiperazine-2,5-dione (**220**) 3-Isobutyl-6-sec-butyl-piperazine-2,5-dione (**221**)	*Pyricularia oryzae*	Induce morphological deformation of mycelia germinated from conidia of *Pyricularia oryzae*	*Alternaria* sp.		[58]
Pyrophen (**103**)	% Inhibition of Brine shrimp	4–11% at 34.8 μM	*Alternaria alternata* D2006	Soft coral *Denderonephthya hemprichi*	[36]
Rubrofusarin B (**104**)		4–11% at 34.9 μM			
Fonsecin (**105**)		4–11% at 36.4 μM			
Fonsecin B (**106**)		4–11% at 32.8 μM			
Aurasperone A (**107**)		4–11% at 17.5 μM			
Aurasperone B (**108**)		4–11% at 16.5 μM			
Aurasperone C (**109**)		4–11% at 16.8 μM			
Aurasperone F (**110**)		4–11% at 17.4 μM			
Amibromdole (**223**)	Antilarval Settlement Activity	EC_50_ = 35.9 μM	*Alternaria* sp.	Coral	[59]
Altersolanol L (**22**)		EC_50_ = 154.3 μM			
Altersolanol C (**21**)		EC_50_ = 78.1 μM			
Physcion (**224**)		EC_50_ = 176.0 μM			
Tricycloalternarene 3a (**59**)	Marine pathogenic *Vibrio anguillarum*	8 mm at 100 μg/disk	*Alternaria tenuissima* EN-192	Mangrove *Rhizophora stylosa*	[60]
Djalonensone (**231**)		9 mm at 100 μg/disk			
Macrosporin (**38**)	Marine pathogenic *Vibrio anguillarum*	Antimicrobial activity	*Alternaria* sp. WZL003	Gorgonian *Echinogorgia rebekka*	[61]
(±)-(4S*,5S*)-2,4,5-Trihydroxy-3-methoxy-4-methoxycarbonyl- 5-methyl-2-cyclopenten1-one (**123**)	*Fusarium graminearum*, *Calletotrichum musae*	MIC = 215.5, 862.0 μM	*Alternaria* sp. R6	Mangrove *Myoporum* *bontioides*	[41]
Fischexanthone (**124**)		MIC = 474.6, 474.6 μM			
4-Chloro-1,5-dihydroxy-3-hydroxymethyl-6-methoxycarbonyl-xanthen-9-one (**159**)		MIC = 107.1, 214.2 μM			
Sesteralterin (**232**)	Three marine phytoplankton (*Chattonella marina*, *Heterosigma akashiwo*, *Prorocentrum donghaiense*), one marine zooplankton (*Artemia salina*); marine-derived bacterium (*Pseudo-alteromonascitrea*)	41–69% inhibition of three marine phytoplankton	*Alternaria alternata* k21-1	Red alga *Lomentaria* *hakodatens*	[62]
Tricycloalterfurene A (**233**)		64, 37, 46% inhibition of three marine phytoplankton			
Tricycloalterfurene B (**234**)		Weak to moderate at 264.5 μM, more sensitive to *C. marina*			
Tricycloalterfurene C (**235**) Tricycloalterfurene D (**236**)		17–56% inhibition of three marine phytoplankton			
TCA-F (**237**)		70, 41, 52% inhibition of three marine phytoplankton			
(2*E*)-TCA 12a (**238**)	Marine phytoplanktons (*Chattonella marina*, *Heterosigma akashiwo*, *Prorocentrum donghaiense*); one marine zooplankton (*Artemia salina*)	3.6–48.6%	*Alternaria alternata* k23-3	Red alga *Gelidiella acerosa*	[63]
(2*Z*)-TCA 12a (**239**)		23.8–79.6%			
TCA 11a (**240**)		23.5–51.6%			
17-*O*-methyltricycloalternarene D (**241**) Methyl-nortricycloalternarate (**242**) TCA 1b (**243**)	Four marine plankton species (*Chattonella marina*, *Heterosigma akashiwo*, *Prorocentrum donghaiense*); marine zooplankton (*Artemia salina*)	Weak or moderate activity	*Alternaria alternata* k21-1	Red alga *Lomentariaha* *kodatens*	[64]
TCA D (**244**)		78.5% of *C. marina* at 256.4 μM Less toxic to *A. salina* 12.6%			
Stemphyperylenol (**55**)	*A. alternata* (Fries) *Keissler*, *A. brassicicola*, *P. parasitica var. nicotianae* Tucker, *D. medusaea* Nitschke, *A. niger* van. Tiegh, *P. theae*	MIC = 22.1, 355.1 µM (*P. theae*, *A. brassicicola*)	*Alternaria* sp. P8	Marine plants	[65]
Alterperylenol (**68**)		MIC = 5.57 µM (*C. michiganensis*)			
(+)-(2*S*,3*R*,4a*R*)-Altenuene (**245**)	Phytotoxicity against seed germination and seedling growth of amaranth and lettuce *A. alternata* (Fries) Keissler, *A. brassicicola*, *D. medusaea* Nitschke, *P. theae* Three plant pathogenic bacteria, *A. avenae*, *P. syringae* pv. *lachrymans*, and *R. solanacearum*	Phytotoxicity against the seedling growth of amaranth and lettuce at 200 ppm *A. brassicicola*, MIC = 428.0 μM	*Alternaria* sp. P8	Seawater, sediments, animals, and algae	[66]
(+)-Isoaltenuene (**246**)		Phytotoxicity against the seedling growth of amaranth and lettuce at 200 ppm			
Altertoxin I (**52**)		Phytotoxicity against the seedling growth of amaranth and lettuce at 200 ppm MIC = 177.5 μM (*D. medusaea*)			
Stemphyperylenol (**55**)		Phytotoxicity against the seedling growth of amaranth and lettuce at 200 ppm			
Alterperylenol (**68**)		Phytotoxicity against the seedling growth of amaranth and lettuce at 200 ppm			
Alterbutenolide (**247**)	*Chattonella marina*, *Heterosigma akashiwo*, *Prorocentrum donghaiense*, *Artemia salina*	IC_50_ = 144.1 μM (*C. marina*)	*Alternaria alternata* I-YLW6-1	Sponge	[67]
Alternariol (**41**)		IC_50_ = 11.6–140.3 μM			
Asperigillol B (**248**)		IC_50_ = 31.2–88.9 μM LC_50_ > 367.6 μM (*A. salina*)			
*p*-Hydroxyphenylacetic acid (**249**)		LC_50_ > 657.8 μM (*A. salina*)			
16-Methoxy solanapyrone B (**254**)	Herbicidal against weed *E. crusgalli* Antifungal against soil-borne *B. cinerea* from grape (BCG) and strawberry (BCS) *F. oxysporum f*. sp. *cucumerinum* (FOC) and *F. oxysporum f*. sp. *Lycopersici* (FOL)	43.1–61.3% at 125.7 µM (*E. crusgalli*)	*Alternaria iridiaustralis*	*Suaeda glauca* plant	[68]
Solanapyrone B (**255**)		43.1–61.3% at 131.5 µM (*E. crusgalli*)			
Solanapyrone S (**256**)		43.1–61.3% at 106.3 µM (*E. crusgalli*)			
Probetaenone I (**257**)		43.1–61.3% at 125.0 µM (*E. crusgalli*)			
Alternanone A (**258**)		MIC = 214.7 µM (*B. cinerea* both strains)			
Alternanone B (**259**)		60.3% and 72.6% inhibition rate at 98.0 and 196.0 µM (*E. crusgalli)*; MIC = 313.7 µM (*B. cinerea* both strains); MIC = 627.4 and >1000 µM (*F. oxysporum* (FOC) and (FOL))			
Chaetosemin D (**260**)		MIC > 1000 µM (*F. oxysporum* (FOC) and (FOL))			
Alternanone C (**261**)		MIC = 141.5 µM (*B. cinerea* both strains); MIC = 566.3 and >1000 µM (*F. oxysporum* (FOC) and (FOL))			
Tenuazonic acid (**89**)		90% inhibition rate at 101.5 and 203.0 µM against *E. crusgalli*			

Note: Phytotoxic activities are listed as IC_50_, LC_50_, MIC in μM or as % of inhibition where available. IC_50_, LC_50_, MIC values for all compounds have been converted to μM for consistency.

### 2.8. Compounds with Miscellaneous Activities (Table 8)

**Figure 18:**
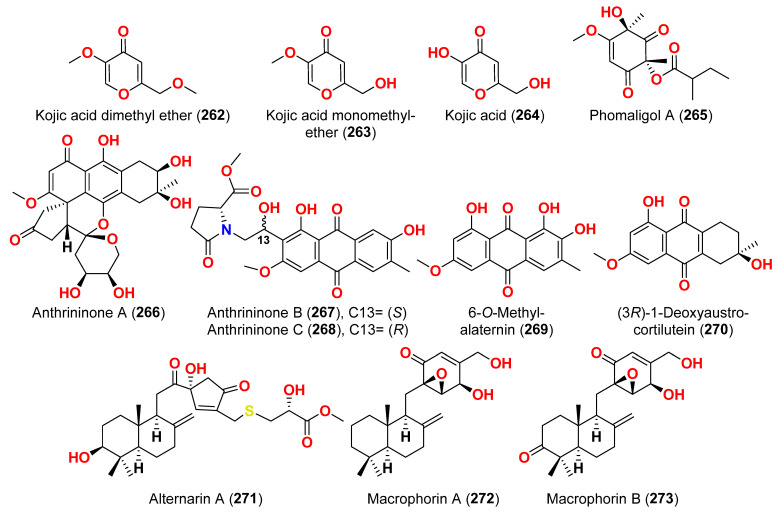
Structures of compounds **262**–**273**.

Compounds evaluated for their miscellaneous effects are displayed in Figure 1, Figure 2 and Figure 18. Some isolated marine natural products have served as potential lead compounds for clinically useful drugs and have been used as chemical probes for fundamental studies in life science. Kojic acid dimethyl ether (**262**), kojic acid monomethyl ether (**263**), kojic acid (**264**), and phomaligol A (**265**) were isolated from marine-derived fungus *Alternaria* sp. MFA 898, which was collected from the green algae *Ulva pertusa* on Jeju Island, Korea [69]. Spectrophotometric analysis was used to determine the antityrosinase activity of the compounds. Among them, kojic acid (**264**) was found to have significant antityrosinase activity with an IC_50_ value of 12.0 µM. However, the remaining compounds were found to be inactive [69]. Sg17-1-4 (**3**) was yielded from the fungus *Alternaria tenuis* Sg17-1-4, which was isolated from a marine alga collected on Zhoushan Island, China [8]. Sg17-1-4 (**3**) is an isocoumarin with a seven-numbered ring on its side chain, and displayed anti-ulcer activity [8].

The hydroanthraquinone—anthrininone A (**266**)—two anthraquinones—anthrininones B and C (**267** and **268**)—in addition to 6-*O*-methylalaternin (**269**), were obtained from the fungus *Alternaria tenuissima* DFFSCS013, which was isolated from a deep-sea sediment, collected from the South China Sea [70]. Compounds **266**–**269** had significant inhibition against indoleamine 2,3-dioxygenase 1 (IDO1), especially anthrininone C (**268**), with an IC_50_ value of 0.5 μM. Furthermore, compounds **267**–**269** exhibited selective inhibitory activities against five different protein tyrosine phosphatases (PTPs), including TCPTP, SHP1, MEG2, SHP2, and PTP1B. The IC_50_ value of compound **269** for PTP1B was 2.1 μM, which was 17.1- and 14.3-fold less than that for TCPTP (IC_50_ = 35.3 μM) and PTP-MEG2 (IC_50_ = 29.6 μM), respectively. The anthraquinone moiety is necessary for enzymatic inhibition, and the substituents on the structure can influence the observed activity. Moreover, compounds **266**–**269**, together with (3*R*)-1-deoxyaustrocortilutein (**270**), dihydroaltersolanol A (**18**), altersolanol L (**22**), ampelanol (**23**), and altersolanol B (**20**) were evaluated for their effects on intracellular calcium flux in HEK293 cells using a calcium imaging assay. Compound **266**, at 10 μM concentration, stimulated intracellular calcium levels, and its fluorescence count was around 25% of calcium ionophore I (CA 1001). However, compound **266** at <10 μM concentration and other compounds at 10 μM concentration did not affect intracellular calcium levels [70].

Alternarin A (**271**) was isolated together with two analogs, macrophorins A (**272**) and B (**273**), from the fungus *Alternaria* sp. ZH-15, which was isolated from a *Lobophytum crassum* soft coral collected in the South China Sea [71]. The isolated meroterpenoids were evaluated for their neuronal modulatory activity by testing the effect on spontaneous Ca^2+^ oscillations (SCOs) in primary cultured neocortical neurons. Alternarin A (**271**) concentration-dependently inhibited SCOs by decreasing both the spontaneous SCO frequency (IC_50_ = 3.2 μM) and amplitude (IC_50_ = 1.8 μM). Furthermore, compound **271** also effectively suppressed hyperactive SCOs induced by the seizurogenic agent 4-aminopyridine (4-AP) in cortical neurons, with IC_50_ values of 10.0 μM for frequency and 4.6 μM for amplitude, respectively, indicating its inhibitory activity on neuronal excitability. Compounds **272** and **273** were inactive in modulating SCOs in neocortical neurons. The results showed that the reorganized meroterpenoid drimane (**271**), with an unusual cyclopentenone moiety, is a novel neuroactive compound with potential anti-epileptic properties [71].

The results shown above support the miscellaneous effect of marine *Alternaria*-derived fungal metabolites. Kojic acid (**264**) showed significant antityrosinase activity with IC_50_ value of 12.0 µM. Conversely, the analogs, kojic acid dimethyl ether (**262**) and kojic acid monomethyl ether (**263**), were inactive [69], suggesting the importance of the free hydroxyl group for enzymatic inhibition. Within the anthraquinone class, anthrininone C (**268**) demonstrated strong inhibition of IDO1 (IC_50_ = 0.5 µM), while 6-*O*-methylalaternin (**269**) selectively inhibited PTP1B (IC_50_ = 2.1 µM), with reduced activity against other PTPs [70]. These data suggest that the anthraquinone core is crucial for activity, and specific substituents modulate both potency and target selectivity. In the meroterpenoid drimane series, alternarin A (**271**) displayed potent neuronal modulatory activity, suppressing spontaneous Ca^2+^ oscillation frequency (IC_50_ = 3.2 µM) and amplitude (IC_50_ = 1.8 µM), as well as 4-AP-induced hyperactive oscillations, while its analogs, macrophorins A and B (**272** and **273**), were inactive [71], highlighting the functional importance of the cyclopentenone moiety.

Moderately active compounds in this group are represented by anthrininone A (**266**), which induces intracellular calcium flux at 10 µM, and Sg17-1-4 (**3**) which exhibits anti-ulcer activity [8,70]. These compounds, while less potent, still provide insight into functional group contributions to bioactivity.

Overall, structure–activity relationship analyses indicate that minor modifications, such as methylation, hydroxylation, or ring rearrangements, dramatically influence biological outcomes. For antityrosinase activity, free hydroxyl groups are required for copper chelation; for anthraquinones, the planar core is essential for enzyme binding, with substituents governing selectivity; and for meroterpenoid drimanes, the cyclopentenone ring is critical for neuroactivity. These observations underscore the potential of marine fungi as a source of structurally diverse bioactive compounds and provide a framework for the rational design of more potent and selective analogs. Compounds of reported miscellaneous activities are shown in Table 8.

**Table 8:** Reported compounds showing miscellaneous activities.

Compound	Applied Assay	Biological Activity	Fungus Name	Host Organism	Reference
Kojic acid (**263**)	Tyrosinase inhibitory activity	IC_50_ = 12.0 μM	*Alternaria* sp. MFA 898	The green alga *Ulva pertusa*	[69]
Sg17-1-4 (**3**)	Anti-ulcer activity	Active	*Alternaria tenuis*, Sg17-1	Unspecified marine alga	[8]
Anthrininone A (**266**)	Inhibition activity against five (PTPs), Indoleamine 2,3-dioxygenase 1 (IDO1) Effects on intracellular calcium flux	Significant inhibition activity against IDO1 Stimulate intracellular calcium levels at 10 μM	*Alternaria tenuissima* DFFSCS013	Deep sea sediment	[70]
Anthrininone B (**267**)		Significant inhibition activity against IDO1 Selective inhibition activity against five PTPs			
Anthrininone C (**268**)		Selective inhibition activity against five PTPs Significant inhibition activity against IDO1, IC_50_ = 0.5 μM			
6-*O*-Methylalaternin (**269**)		Significant inhibition activity against IDO1 Selective inhibition activity against five PTPs, IC_50_ for PTP1B = 2.1 μM			
Alternarin A (**271**)	Neuronal modulatory activity by testing the effect on spontaneous Ca^2+^ oscillations (SCOs) in primary cultured neocortical neurons	IC_50_ = 3.2 μM against SCO frequency IC_50_ = 1.8 μM against amplitude	*Alternaria* sp. ZH-15	Soft coral *Lobophytum* *crassum*	[71]

## 3. Secondary Metabolites Which Were Not Evaluated for Their Bioactivities

Several compounds have been reported from *Alternaria* without associated biological reports or data confirming their biological effects, representing promising candidates for future pharmacological or ecological investigations. Numerous *Alternaria* metabolites, particularly those reported for novel structures, were thus not evaluated in bioassays by the original authors. These compounds remain as chemical entities of undetermined bioactivity—potentially inactive or active beyond standard screening panels (e.g., enzyme inhibition or ecological signaling). While previous versions of this review highlighted their possible “biomedical potential,” we acknowledge that such claims are speculative without empirical validation. Therefore, these compounds are best regarded as chemically intriguing but biologically uncharacterized. Rather than asserting biological promise, we emphasize their research value as untested metabolites warranting systematic evaluation. Broader bioassay profiling, including anti-inflammatory, antiviral, and antiparasitic screens or modern high-throughput approaches, could determine whether any possess genuine pharmacological or ecological relevance. Systematic evaluation of these compounds is a priority for elucidating the full functional diversity of the *Alternaria* metabolome.

In addition to their prospective biological significance, inclusion of these metabolites is chemically and taxonomically valuable. Each untested compound contributes to defining the chemical diversity and biosynthetic capacity of the genus *Alternaria*, information that is crucial for chemotaxonomic classification and comparative studies among related genera. The structural diversity, spanning ceramides, benzopyranones, anthraquinones, and polyketides, reflects lineage-specific metabolic pathways that can support taxonomic differentiation and phylogenetic mapping. Thus, documenting these compounds in this review, even in the absence of known bioactivity, enriches the chemical framework necessary for understanding *Alternaria* systematics and evolutionary relationships.

These compounds include cerebroside C (**274**), bisdethiobis(methylthio)acetylaranotin (**275**), cerebroside D (**276**), acetylaranotin (**277**), *N*-acetyltyramine (**278**), cyclo-(Tyr-Pro), (22*E*,24*R*)-3β,5α-dihydroxy-23-methylergosta-7,22-dien-6-one (**279**), (22*E*,24*R*)-3β,5α,9α-trihydroxyergosta-7,22-dien-6-one, (22*E*,24R)-23-methylergosta-7,22-diene-3β,5α,6β-triol (**280**), cerevisterol (**281**), 6β-methoxyergosta-7,22-diene-3β,5α-diol (**282**), ergosterol peroxide (**283**), and ergosterol (**284**), which are reported from the marine-derived fungus *Alternaria raphani* [9]. Similarly, the mangrove-endophytic *Alternaria* sp. ZJ9-6B yielded alterporriol M (**285**) and dactylariol (**286**) [11]. Compounds from *Alternaria brassicae* 93, including ochratoxin A methyl ester (**287**), cis-4-hydroxymellein (**288**), (R)-7-hydroxymellein (**289**), trans-2-anhydromevalonic acid (**290**), and protocatechuic acid (**291**), were likewise untested [21]. Additionally, the compounds pachybasic acid (**292**), emodic acid (**293**), emodin (**294**), phomarin (**295**), and 1,7-dihydroxy-3-methylanthracene-9,10-dione (**296**) are reported from a fish-derived fungus *Alternaria* sp. X112, and are without biological reports [31]. The sea cucumber-derived *Alternaria* sp. (HS-3) afforded 4-acetyl-5-hydroxy-3,6,7-trimethylbenzofuran-2(3H)-one (**297**), 2-carboxy-3-(2-hydroxypropanyl)phenol (**298**), and 5-methyl-6-hydroxy-8-methoxy-3-methylisochroman (**299**), also without reported bioactivity [72]. Likewise, *Alternaria alternata* HK-25 yielded 12,13-dihydroxy-fumitremorgin C (**300**), gliotoxin (**301**), demethoxyfumitremorgin C (**302**), bisdethiobis(methylthio)gliotoxin (**303**), and fumitremorgin C (**304**), with no activity data [73]. Investigation of the beach-derived *A. alternata* (Fr.) Keissl. revealed tricycloalternarene 18c (**305**), mannitol (**306**), allantoin (**307**), thymine (**308**), uracil (**309**), erythritol (**310**), and ergosterol (**284**), without recorded bioactivities [74]. Finally, GC–MS analysis of *A. alternata* extracts identified acetic acid ethyl ester (**311**), N-(4,6-dimethyl-2-pyrimidinyl)-4-(4-nitrobenzylideneamino)benzenesulfonamide (**312**), oxiraneundecanoic acid, 3-pentyl-, methyl ester, cis (**313**), hexadecanoic acid (**314**), (Z,Z)-9,12-octadecadienoyl chloride (**315**), (Z)-9-octadecenoic acid (**316**), octadecanoic acid (**317**), phthalic acid di(2-propylpentyl) ester (**318**), and 1,2-benzenedicarboxylic acid (**319**) [75].

## 4. Discussion

**Figure 19:**
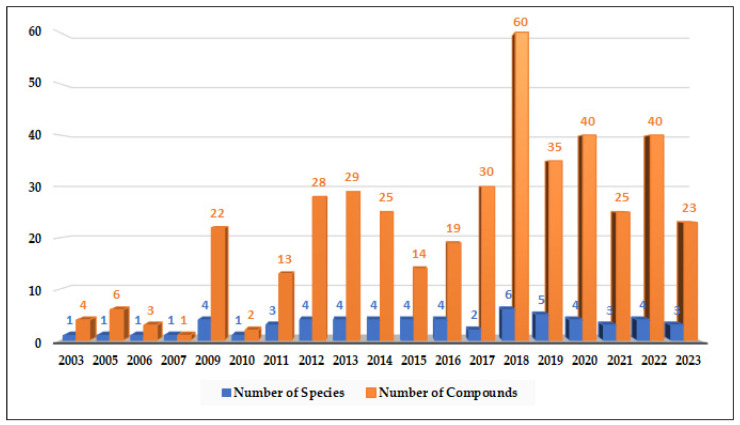
Annual number of investigated *Alternaria* species and reported compounds from these species (2003–2023).

**Figure 20:**
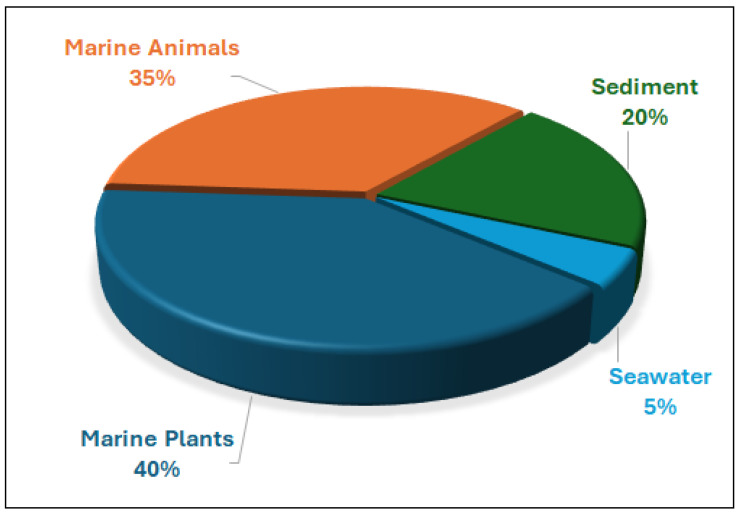
Source of marine fungal *Alternaria* isolates discussed in this report.

**Figure 21:**
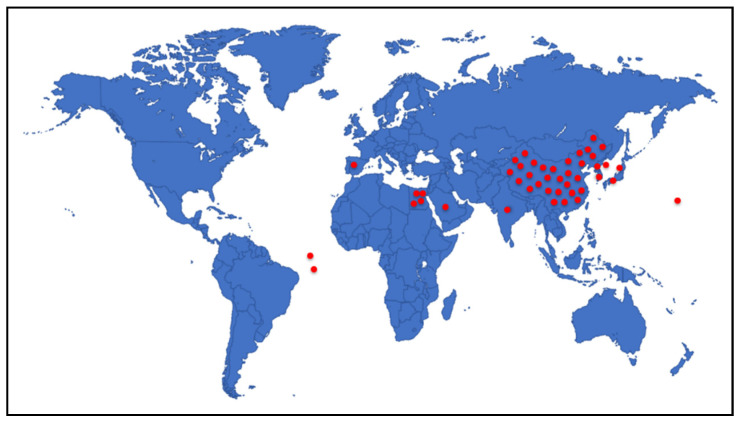
A map showing collection sites of marine-derived *Alternaria* species discussed in this review. Red dots on the map representing countries or locations where the host of *Alternaria* fungi reported in this review was harvested.

This review provides an updated report of 319 natural products reported from 67 marine-derived *Alternaria* species, studied between 2003 and 2023 (Figure 19). These fungi were isolated from diverse marine sources, including plants, animals, sediments, and seawater, reflecting their broad ecological adaptability in various aquatic environments (Figure 20). The predominance of fungal isolates from Chinese marine territories (~79%) highlights both the research strength in this region and the need for expanded global exploration of members of *Alternaria* worldwide (Figure 21).

**Figure 22:**
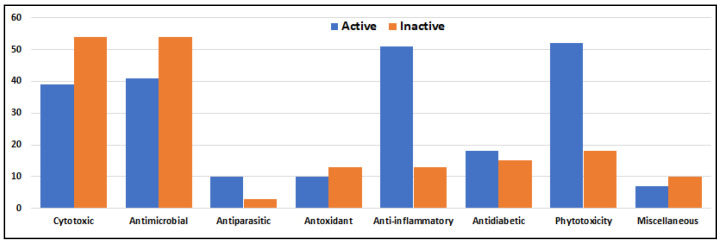
Active versus inactive compounds are evaluated in different screening platforms.

**Figure 23:**
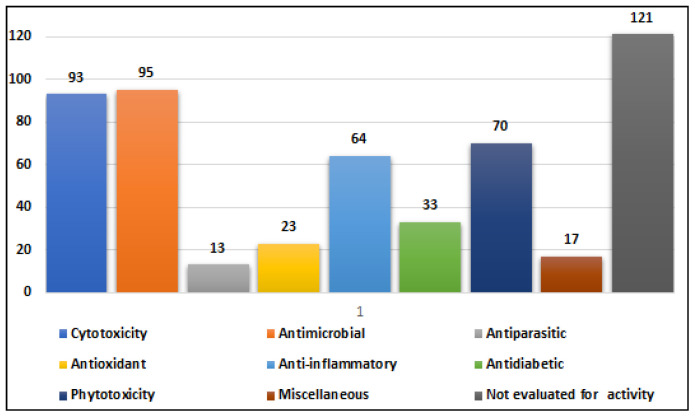
Number of compounds evaluated in different screening platforms.

Of the metabolites assessed in diverse screening assays, roughly 56% displayed one or more detectable activities in one or more screening platforms. The predominant activities were anti-inflammatory (51 compounds), antimicrobial (41 compounds), cytotoxic (39 compounds), and phytotoxic (52 compounds). Furthermore, metabolites with antiparasitic, antidiabetic, and antioxidant activities were also reported (Figure 22 and Figure 23). It is also worth to mention that; some compounds display different activities in different screening platforms suggesting broad-spectrum bioactivity.

Below, we summarize two decades of research on *Alternaria*-derived compounds, outlining their notable structural diversity, their ecological roles, and evolving SAR insights, and emphasizing the importance of standardized, mechanism-oriented investigations to unlock their full biomedical and biotechnological promise.

### 4.1. Overview of Key Bioactive Compound Classes

Research over the past two decades has revealed several recurring structural scaffolds that serve as the primary bioactive agents among *Alternaria*-derived metabolites. Among these, perylenequinones, such as altertoxins I (**52**) and II (**88**), stemphyperylenol (**55**), and alterperylenol (**68**), stand out as some of the most potent cytotoxins, exhibiting sub- to low-micromolar EC_50_ or IC_50_ values against a range of human cancer cells, including A549, PC3, HepG2, HCT-116, and MCF-7. Their polycyclic, conjugated frameworks act as photodynamic toxins capable of creating reactive oxygen species (ROS), which leads to lipid peroxidation and DNA damage [32]. Anthraquinone dimers, exemplified by the alterporriols, display moderate to strong cytotoxicity with distinct selectivity profiles; for instance, alterporriol L (**12**) induces 86% cell death in MCF-7 cells at 50 μM, while alterporriol P (**13**) exhibits IC_50_ values between 6.4 and 8.6 μM against PC-3 and HCT-116 cell lines. Tetramic acid derivatives, including tenuazonic acid (**89**) and ACTG-toxins D and H (**62** and **161**), are predominantly phytotoxic yet demonstrate additional mild cytotoxic and antiviral properties. Tenuazonic acid (**89**), for example, inhibits plant growth by over 90% at concentrations of 101–203 μM through the disruption of photosystem II electron transport and induction of ROS accumulation. Meanwhile, cyclic peptides and nitrogenous compounds such as alternaramide (**99**) and 2,4,6-triphenylaniline (**217**), though less commonly encountered, hold notable pharmacological relevance: alternaramide acts as a PTP1B inhibitor with potential antidiabetic implications, while 2,4,6-triphenylaniline exhibits moderate dual α-amylase and α-glucosidase inhibition, suggesting metabolic regulatory potential. Collectively, these structural classes illustrate the remarkable chemical diversity and mechanistic versatility of *Alternaria* metabolites, reinforcing their promise as leads for drug and agrochemical development.

### 4.2. Biomedical vs. Ecological Perspectives

*Alternaria*-derived compounds often serve ecological roles, such as phytotoxic compounds aiding plant colonization or antimicrobial agents defending against microbial competitors. Cytotoxic compounds may protect fungal spores from UV damage, while phytotoxic and antifungal compounds align with herbicidal and crop-protective applications. Recognizing these natural functions aids in repurposing metabolites for biomedical and agricultural use.

It is crucial to distinguish between the biomedical applications of these compounds and their ecological functions. Many of these compounds likely evolved to confer ecological advantages. For example, phytotoxic compounds such as tenuazonic acid and altertoxin derivatives help the fungus colonize or kill plant tissue, acting as host-specific toxins in plant disease [6]. Similarly, antimicrobial compounds, including alternariol derivatives and perylenequinones, likely serve as defense molecules, suppressing bacterial or fungal competitors in the marine environment [76].

Understanding these natural roles can guide practical applications. For instance, phytotoxic metabolites may be repurposed as eco-friendly herbicides or algaecides, aligning agricultural use with their ecological function. Conversely, pigmented compounds, such as perylenequinones, may absorb UV radiation, protecting spores in natural settings. Their cytotoxicity toward mammalian cells could be incidental, yet it can be harnessed for anticancer drug discovery. Similarly, ROS-generating or enzyme-inhibitory metabolites may simultaneously defend the fungus, mediate interspecies interactions, and offer therapeutic leads. Thus, the ecological context provides insights into the functional versatility of these metabolites and informs rational biotechnological exploitation.

### 4.3. Challenges in Data Comparability

Comparing bioactivity data across studies remains challenging due to the lack of standardized assay protocols. Different laboratories used varied cell lines, concentrations, incubation times, and reference compounds, which can substantially affect reported potency. For example, an IC_50_ of 10 μM in one study might not be directly comparable to the same value in another if the assay duration, readout method, or cell type differs.

To improve comparability, we normalized units and noted reference compounds, such as cisplatin for cytotoxicity, or ampicillin for antibacterial activity. However, caution is still warranted. Standardization, such as reporting cytotoxicity using the MTT assay at 72 h with a common drug standard, would facilitate more reliable cross-study analyses. Without uniform protocols, subtle structure–activity relationships or selectivity patterns might be obscured, potentially slowing the identification of promising lead compounds.

### 4.4. Inactive and Untested Compounds—Future Opportunities

Approximately 44% of the reported metabolites had no documented bioactivity. This is common in natural product research, where initial studies focus on structural elucidation rather than functional evaluation. However, it indicates a considerable untapped resource of potentially bioactive molecules.

Future research should systematically screen these “orphan” compounds across broader assay panels. For example, antioxidant compounds like altenusin might confer neuroprotective effects, while genotoxic compounds such as alternariol could inhibit viral polymerases. Identifying their activities could reveal new drug leads or confirm truly inactive scaffolds, optimizing resource allocation. Recognizing this knowledge gap emphasizes the need for comprehensive, multitarget bioassays to fully capture the biomedical and ecological potential of *Alternaria*-derived molecules.

### 4.5. Misidentification Risks

Chemical structure accuracy is critical when evaluating bioactivities. While we relied on the structures as reported, complex molecules, especially those with multiple stereocenters, may be misassigned. The fungal metabolite literature includes examples of stereochemical reassignment due to limited NMR or NOE data.

Although we found no specific corrections for marine-derived *Alternaria* compounds during 2003–2023, future studies should employ rigorous structure-determination methods, including X-ray crystallography and electronic circular dichroism (ECD) calculations, to confirm absolute configurations. Accurate structural assignments are essential for SAR studies, mechanistic elucidation, and semisynthetic modification.

### 4.6. Structure–Activity Relationships and Selectivity

Two decades of data on marine *Alternaria*-derived compounds reveal key SAR trends:

- Perylenequinones: Functional groups such as epoxides (e.g., altertoxin II) can slightly reduce cytotoxicity compared with unmodified analogs (altertoxin I), potentially by altering ROS generation or DNA intercalation efficiency.
- Diphenyl ethers (altenuene, altenusin): Phenolic hydroxyl groups enhance antifungal activity, whereas dimethylation reduces efficacy, suggesting that hydrogen-bonding interactions with targets are critical.
- Alternariol derivatives: Small methylation changes can tune cytotoxicity and selectivity; for instance, alternariol preferentially inhibits HL-60 leukemia cells while sparing normal lymphocytes.
- Perylenequinone and anthraquinone dimers: Planarity and free hydroxyl groups correlate with antiparasitic and antioxidant potency. Methylation or dimerization can decrease activity, indicating steric and electronic effects on target interaction.
- Enzyme inhibitors: Hydroxylation at specific positions enhances α-glucosidase, α-amylase, or PTP1B inhibition, guiding rational derivatization for antidiabetic applications.
- Phytotoxic compounds: ROS-mediated photosystem disruption is central, and planar quinone/perylene scaffolds correlate with herbicidal potency.

These SAR insights not only inform chemical modification strategies but also enhance understanding of the molecular mechanisms underlying *Alternaria* metabolite bioactivities. Future mechanistic studies should investigate precise enzyme targets, signaling pathways, and cellular uptake differences to fully exploit these natural products in drug discovery, agriculture, and biotechnology.

## 5. Conclusions and Future Trends

Marine-derived fungi of the genus *Alternaria* represent a prolific yet still insufficiently explored source of chemically and biologically diverse secondary metabolites. Over the past two decades, 67 marine-derived *Alternaria* species have been documented, producing over 300 structurally unique compounds. Among these, approximately 56% exhibit one or multiple measurable bioactivities, including anti-inflammatory, antimicrobial, antiparasitic, cytotoxic, antidiabetic, and phytotoxic effects. Notably, key compound classes such as perylenequinones, anthraquinones, tetramic acids, and nitrogenous peptides have emerged as particularly bioactive, with distinctive mechanisms of action ranging from ROS-mediated cytotoxicity to enzyme inhibition and phytotoxicity. These findings underscore *Alternaria* as a valuable reservoir of bioactive molecules with potential applications across pharmaceuticals, agriculture, and biotechnology.

Our analysis also highlights significant gaps and biases in current research. Geographically, approximately 79% of the isolates in this review were from Chinese marine environments, revealing a clear regional bias and signaling the need for broader sampling from underexplored habitats worldwide, including deep-sea sediments, polar regions, and coral-associated niches. Structurally, nearly half of the reported compounds (≈44%) have not been evaluated for bioactivity, representing a largely untapped reservoir of potentially novel pharmacophores. Methodologically, the lack of standardized screening protocols, ranging from inconsistent assay conditions to diverse definitions of “activity”, limits data comparability and the ability to accurately prioritize compounds for further study.

To address these challenges, future research should adopt more targeted strategies including:

(1) Geographically and ecologically diversified sampling. Systematic exploration of underrepresented regions and unique ecological niches is likely to reveal novel species and metabolites. Priority should be given to habitats that differ chemically and physically from previously sampled regions, as they may drive the production of unique secondary metabolites.
(2) Focused compound-class investigations. Perylenequinones, anthraquinones, and tetramic acids have repeatedly demonstrated potent bioactivities and deserve mechanistic and SAR-focused studies. For example, perylenequinones show light-dependent ROS generation, making them promising leads for anticancer and antimicrobial therapies. Similarly, tetramic acids such as tenuazonic acid, while primarily phytotoxic, also show antiviral and moderate cytotoxic activity and could be explored as multifunctional agents.
(3) High-throughput and omics-integrated discovery. Integrating genomics, transcriptomics, and metabolomics can uncover cryptic biosynthetic gene clusters and activate silent pathways. Co-culture approaches, environmental stress induction, and heterologous expression can further enhance metabolite yields and structural diversity.
(4) Enhanced screening and standardization. Establishing uniform bioassay protocols, including common reference compounds and standardized endpoints, will improve cross-study comparability. Expanding bioactivity testing to include antiviral, neuroprotective, and metabolic targets could reveal hidden potential in currently “inactive” compounds.
(5) Translational and ecological insights. Linking compound activity to ecological function can guide applied uses. For instance, naturally phytotoxic metabolites may be repurposed as environmentally friendly herbicides, while antimicrobial compounds could inform marine biocontrol strategies. Detailed SAR and mechanistic studies will also facilitate semisynthetic optimization for therapeutic purposes.
(6) Accurate taxonomy and reproducibility. Ensuring precise species identification through ITS barcoding, voucher specimens, and phylogenetic analysis is critical to correctly attributing metabolites, reducing misidentification, and supporting reproducible research.

In summary, marine-derived *Alternaria* species constitute a rich but incompletely characterized reservoir of secondary metabolites. Advancing the field will require strategically addressing the current geographical and methodological biases, systematically exploring both well-known and orphan compounds, and applying integrated omics and mechanistic approaches. By combining ecological insights with innovative discovery strategies, future research can unlock the full biosynthetic potential of this genus, driving the development of novel therapeutic agents, sustainable agricultural solutions, and industrially relevant bioactive molecules.

## Data Availability

No new data were created or analyzed in this study. Data sharing is not applicable to this article.
